# A hybrid innovation method based on quality by design and agile scrum paradigms for the development of medicinal products

**DOI:** 10.1038/s41598-025-18181-w

**Published:** 2025-09-29

**Authors:** Thierry Bastogne, Laurène Wagner, Samir Acherar, Gilles Karcher, Charlotte Collet

**Affiliations:** 1 CRAN UMR 7039, CNRS, Université de Lorraine, 54000 Nancy, France; 2 LCPM UMR 7375, CNRS, Université de Lorraine, 54000 Nancy, France; 3 Nancyclotep, 54511 Vandoeuvre-lès-Nancy, France; 4 IADI, INSERM U1254, Université de Lorraine, 54511 Vandoeuvre-lès-Nancy, France

**Keywords:** Quality by design, Agile method, Data-driven innovation, Radiopharmaceutical, Quality control, Diagnostic markers, Data processing

## Abstract

This study proposes an agile paradigm of the pharmaceutical Quality by Design (QbD) approach initiated and recommended by regulatory agencies to better understand and control their innovative products and processes throughout the development phase. The primary objective of this hybrid innovation method is to improve the structural organization of the QbD approach to simplify its use and expand its application to the early stages of preclinical development. This agile QbD paradigm relies on the incrementation and/or iteration of short studies called sprints indexed according to the Technological Readiness Level (TRL) scale. Each QbD sprint addresses a priority question of development and relies on a hypothetico-deductive scientific method to address it. They are composed of five steps: developing and updating the Target Product Profile, identifying critical input and output variables, designing experiments, conducting experiments, and analyzing the collected data to generalize conclusions through statistical inference. At the end of a sprint, four outcomes are possible: incrementing knowledge on the developed drug, i.e. moving to the next development sprint, iterating the current or previous sprint to reduce decision-making risk, pivoting to propose a new product profile, or stopping the development project. This decision-making process is based on the results of a statistical analysis estimating the probability of meeting the efficacy/safety/quality specifications of the medicinal product to be developed. To illustrate and to assess the practical relevance of this incremental and iterative approach, we applied it to the development of a new radiopharmaceutical for Positron Emission Tomography (PET) imaging. The Agile QbD approach was applied over six consecutive sprints to progress from an initial product concept (TRL 2) to a prototype manufactured using a production automation system (TRL 4). The method and results presented in this study provide a new perspective on applying QbD as an efficient tool for managing knowledge during innovation projects.

## Introduction

Drug development is a critical yet complex endeavor that holds immense potential for improving human health, but it is also fraught with high risks, significant costs, and scientific, regulatory, and logistical challenges at every stage. Before the 2000s, pharmaceutical development was primarily managed through the Quality by Testing approach, which relied on the waterfall paradigm, a linear and sequential strategy, where each phase of a project, from concept to completion, must be fully completed before moving on to the next^1^. While it provides a clear structure and control, its rigidity makes it less suited for innovation projects, which often require flexibility and iterative adjustments^2^. It was one of the main reasons that motivated the Food and Drug Administration (FDA) in the early 2000s to promote the Quality by Design (QbD) approach as a proactive paradigm to introduce quality into drug development from the start by understanding how materials and processes affect efficacy and safety outcomes, rather than relying only on final testing^3,4^. Over the past twenty years, QbD has gradually become the best practice for developing new drugs^5–7^ or innovative analytical procedures^8–10^.

We hypothesize that the absence of a well-defined and comprehensive structure within the QbD paradigm may partly explain why this approach is often perceived as complex and challenging to implement. This study aims to address a central question: how can QbD be given a clearer, more explicit framework that enhances knowledge management and supports its broader adoption as a genuine pharmaceutical innovation methodology, starting from the earliest stages of preclinical development.

To rethink the organization of the QbD process, we draw inspiration from innovation models used in other industries, particularly information technology. One such model is the Agile Scrum method, which emerged in the early 1990s as a response to the limitations of traditional linear project management frameworks like the Waterfall model. Scrum offers an iterative and incremental approach to managing innovation, breaking work into short cycles known as sprints, enabling teams to continuously adapt and improve based on regular feedback^11^.

To overcome the previously outlined limitations and establish QbD as a truly effective innovation framework in the pharmaceutical industry, we propose a hybrid model that merges the principles of Quality by Design with the Scrum methodology. This new approach structures the QbD process into systematic, iterative sprints aligned with the Technology Readiness Level (TRL) scale. We provide a scientific characterization of each sprint and a general framework to organize them throughout the development lifecycle. To demonstrate the practical value of this incremental and adaptive strategy, we applied it to the development of a novel radiopharmaceutical intended for Positron Emission Tomography (PET) imaging.

This paper begins with a detailed description of the proposed Agile QbD approach and presents a case study. In a second part, we describe and analyse the application results, before discussing their implications, stressing its limitations, and suggesting research perspectives.

## Methods

**Fig. 1:**
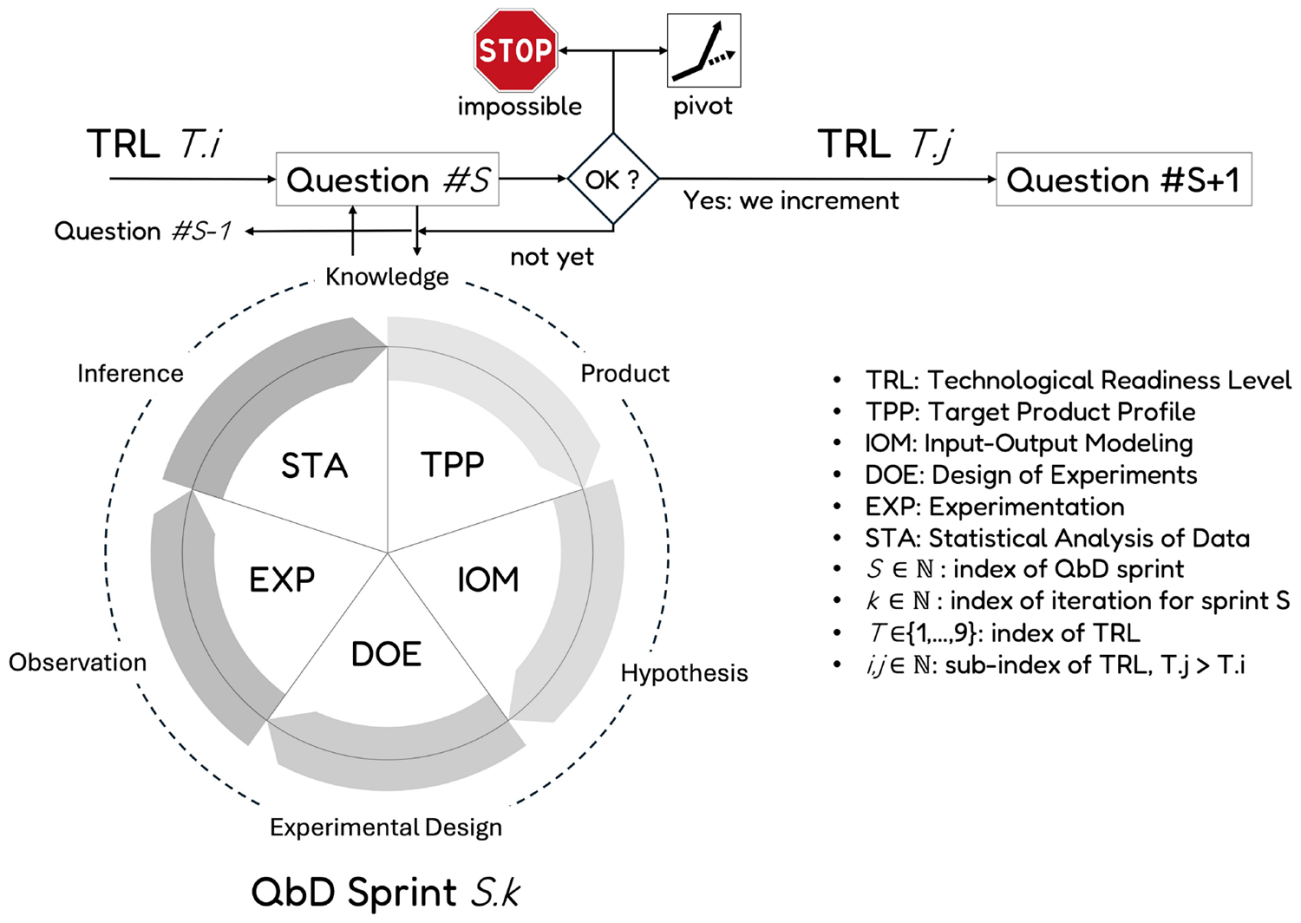
Sprint of the agile QbD innovation paradigm.

This section introduces the complete agile QbD approach developed in this study. Rooted in the Agile Scrum paradigm, it incorporates key structural components such as sprints, user stories, sprint reviews, and backlogs. As shown in Figure 1, each QbD sprint is built around three core elements: a specific development question to be addressed, a corresponding study cycle designed to explore that question, and a qualification test that determines whether the project is ready to advance to the next sprint.

### Questions of investigation

**Fig. 2:**
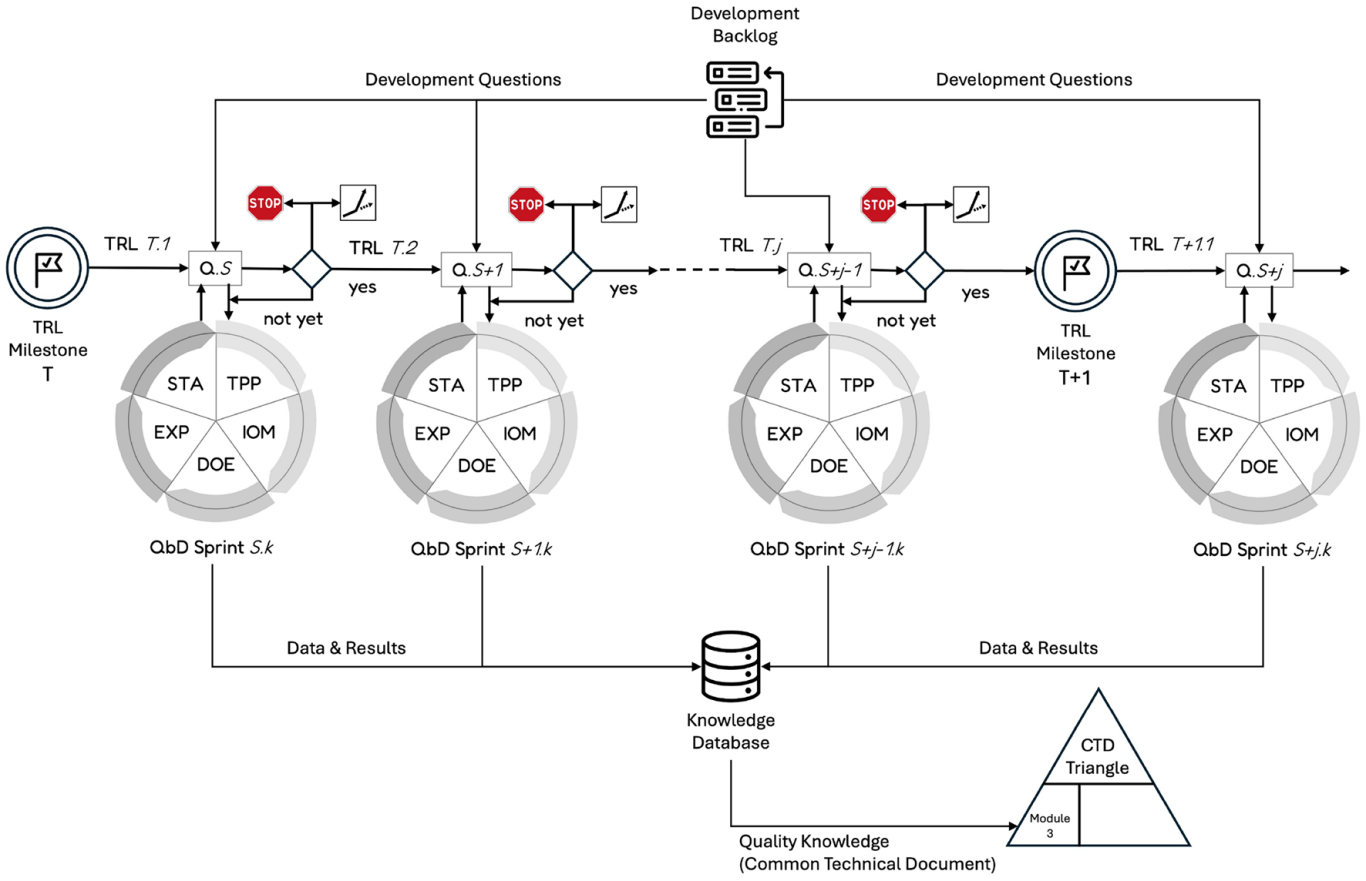
Successive QbD sprints to go from TRL *T* to $T+1$. The knowledge gained through the sprints is structured in a Common Technical Document (CTD). More detailed information on the CTD content is available in^31^.

In Agile methodologies, a user story is a concise, user-centered description of a feature or requirement, written from the end-user’s perspective, outlining their needs and motivations. In the context of the agile QbD approach, this concept is adapted into a structured set of development questions, grouped within a backlog as illustrated in Fig. 2. A real-world example of such innovation-driven questions is presented in Table 2. These development questions are categorized into three distinct types: screening, prediction and qualification issues. These questions can originate from various stakeholders involved in the project, including developers, regulatory authorities, investors, and project managers. To promote consistency and generalizability, we propose the following formats for expressing each type of question:

- Screening question: “As a developer, regulator, manager, or investor, what are the most critical input variables that influence the output variable to be controlled ?”
- Optimization question: “As a developer, regulator, manager, or investor, what is the range of input variables (operating region) that is likely to meet the output specifications considered in this sprint, with an acceptable level of confidence ?”
- Qualification question: “As a developer, regulator, manager, or investor, is the predicted operating region for the targeted output variable sufficiently qualified ?”

Additional investigation questions can be formulated as needed.

### A QbD sprint based on a hypothetico-deductive cycle

To address the previously defined development questions, the proposed investigation engine follows a five-step cycle, as shown in Fig. 1. The structure of this cycle reflects a Hypothetico-Deductive scientific approach^12^. Each cycle begins with a clearly formulated development question, which leads to the generation of hypotheses—here represented as mathematical models and equations. These hypotheses guide the design and execution of experiments, from which observations are collected. The resulting data are then analyzed using statistical inference methods to provide a rigorous and evidence-based response to the original question.

#### Target product profile

The Target Product Profile (TPP) is a strategic document that defines the key attributes, intended use, and development goals of a product, serving as a roadmap that aligns its evolution with regulatory, clinical, and market expectations. It is a dynamic document, refined iteratively after each sprint. For a pharmaceutical product, the TPP typically includes a concise overview covering the indication and target population, formulation, mechanism of action, dosage, non-clinical and clinical outcomes, contraindications, safety considerations, biocompatibility, regulatory pathways, market positioning, intellectual property, and packaging and labeling. It also incorporates commercial objectives, which can be structured using tools like the Business Model Canvas. In the pharmaceutical context, an additional component— the Quality Target Product Profile (QTPP)—is included, detailing the desired pharmacological properties and quality attributes of the drug^13^.

We also recommend incorporating a process mapping component, recognizing that the product and its manufacturing process are intrinsically linked, and that innovation can emerge at the process level as well. The ICH Q9 guideline^14^ outlines several techniques for process decomposition, among which the Process Flow Diagram (PFD) is one of the most widely used tools. This is typically followed by a Failure Modes, Effects, and Criticality Analysis (FMECA) to identify critical manufacturing steps and prioritize unresolved issues that require focused attention^15–18^.

#### Input-output modeling (IOM)

By output variable, we refer to either a Critical Quality Attribute (CQA) or a Key Performance Attribute (KPA)—that is, any variable representing a desired property related to efficacy, safety, or industrial and environmental performance. Input variables encompass all controllable factors, such as material attributes and process parameters, that may influence these output variables. Within the IOM (input-output modeling) step of the QbD sprints, the objective is to formulate hypotheses aligned with the specific development question being addressed. Since there are three primary types of development questions, three corresponding categories of hypotheses must be tested.

1. The first step in hypothesis formulation focuses on identifying the critical input and output variables. To achieve this, the most commonly employed tools in QbD studies include the Cause and Effect Diagram (also known as the Fishbone Diagram), the Process Flow Diagram, and the Failure Modes, Effects, and Criticality Analysis (FMECA)^15–18^. Alternatively, this type of hypothesis can also be expressed mathematically. In the context of a factor screening problem, an affine (linear) model provides a suitable representation:
  1 $$\begin{aligned} Y = b_0 + b_1 x_1 + b_2 x_2 + \cdots + b_px_p + E, \end{aligned}$$
  where *Y* represents the output variable to be controlled, $x_i\in \{0;1\}$ denotes the normalized variable related to the *i*-th input $u_i\in \{A_i;B_i\}$, with $A_i$ and $B_i$ indicating the tested levels during the experiments. *E* accounts for the modeling error, which can be described using a stochastic approach. Most commonly, *E* is modeled as a Gaussian random variable with zero mean and constant variance, i.e., $E\sim \mathcal {N}(0,\sigma ^2)$, although other distributions may also be appropriate depending on the context. The parameter $\sigma$ provides an estimate of the uncertainty associated with the model. The most informative components of this equation, however, are the coefficients $b_i$, as they quantify the influence of each input variable on the output. The greater the absolute value of $b_i$, the more critical the corresponding input factor is for determining *Y*. These coefficients offer a direct means of answering the screening question by identifying which input variables have the most significant effects. This approach is comparable to a first-order sensitivity analysis, which evaluates individual factor effects but does not capture nonlinearities or interactions between inputs. As a result, it may overlook combined or synergistic effects. To address this limitation, more advanced models that incorporate interaction terms can be applied, along with global sensitivity analysis techniques—such as the total sensitivity indices proposed by A. Saltelli et al.^19^—to enable a more comprehensive assessment of factor criticality.
  In our context, the consequences of overlooking a truly critical factor are more severe than those of mistakenly identifying a non-critical one. Therefore, relying solely on the p-value from a significance test on the coefficients $b_i$ is not sufficient for determining the criticality of an input variable. A small p-value does not necessarily indicate a strong or important effect, and conversely, a large p-value does not confirm the absence of relevance or impact. In fact, even large effects may yield weak p-values when sample sizes are limited or measurements are noisy^20^. As in FMECA analyses, the level of uncertainty surrounding a factor’s effect is itself a valuable indicator. When this uncertainty is high, it should be taken into account when assessing the factor’s criticality. For this reason, we generally prioritize examining the 95% confidence intervals of the estimated effects, as they provide a more nuanced and informative basis for selecting critical parameters.
2. The second component focuses on the nature of the cause-and-effect relationships linking input and output variables. As before, these relationships can be more precisely expressed through mathematical modeling, allowing for the formulation of testable hypotheses. Such models may be theoretical, empirical, or a combination of both (e.g., black-box models). A common example is the use of response surface models for addressing empirical optimization problems. These models typically rely on a quadratic polynomial formulation, expressed as follows:
  2 $$\begin{aligned} Y&= \hat{y} + E \end{aligned}$$
  3 $$\begin{aligned} \hat{y}&= b_0+ \sum _{i=1}^{p}b_i\,x_{i}+\sum _{i=1}^{p}b_{ii}\,x_{i}^2+\sum _{i=1}^{p-1}\sum _{j=i+1}^{p}b_{ij}\,x_{i}\,x_{j}, \end{aligned}$$
  where $b_{ii}$ represents the quadratic effect of the *i*-th factor, while $b_{ij}$ captures the interaction effect between the *i*-th and *j*-th factors on the response *Y*. The notation $x_i\in [0;1]$ represents the normalized variable of the *i*-th quantitative input $u_i\in [u_{i,min};u_{i,max}]$, with $u_{i,min}$ and $u_{i,max}$ indicating the exploration bounds of its experimental domain. In such optimization problems, the predicted response $\hat{y}$ is central to the solution. When the prediction error is acceptably low, it becomes possible to estimate the response across the entire experimental space. The resulting response surfaces help identify input regions where predicted outcomes satisfy the desired output specifications. To achieve this, it is also essential to accurately estimate the model coefficients, which requires adhering to a rigorous experimental design methodology, as detailed in the next section.
3. The final category of hypotheses concerns the validity of predictions with respect to meeting the target specifications of the product under development. The objective is to evaluate whether specific operating points—within a defined operating region—produce output values that comply with the required specifications. This hypothesis can be expressed as follows:
  4 $$\begin{aligned} {\left\{ \begin{array}{ll} H_0: Prob\left[ Y\in \Lambda \right] < \pi & \text {The tested operating point does not meet the specifications}\\ H_1: Prob\left[ Y\in \Lambda \right] \ge \pi & \text {It meets the specifications with an acceptable probability: } \pi , \end{array}\right. } \end{aligned}$$
  where the random variable *Y* follows the same probability densitiy function as the one defined in the two previous paragraphs. $\Lambda$ represents the specification limits for the output variable *Y* and $\pi$ denotes the minimum probability threshold defined by the developer.

#### Design of experiments (DOE) & experimentation (EXP)

The results obtained from FMECA studies are often not sufficient to accurately predict the risk of failing to meet the specifications for key output variables, such as Critical Quality Attributes (CQAs) and Key Performance Attributes (KPAs). To improve the reliability of these predictions, the use of mathematical models—whether theoretical or empirical—offers the most effective solution. In both cases, data collection is essential to precisely estimate the model parameters. This is where statistical methods and experimental design tools become indispensable^21^. Within the framework of our agile QbD approach, we highlight four types of experimental designs that are particularly valuable.

The first category includes screening designs, such as Plackett-Burman and definitive screening designs. These experimental approaches are specifically designed to identify the most influential input variables—whether their effects are linear or nonlinear—while minimizing the number of experiments required.

The second category of experimental designs aims to develop behavioral models that describe the relationships between input and output variables. A widely used example of such black-box modeling is the response surface polynomial model^22^, which is particularly effective for addressing empirical response optimization problems. To generate the data needed for building these models, designs such as central composite designs, Box-Behnken designs, Doehlert designs, and Roquemore hybrid designs are especially well-suited.

However, with the rise of machine learning techniques, alternative modeling approaches—such as artificial neural networks, support vector machines, random forests, and more recently, gradient boosted trees—have gained considerable relevance. For these types of models, which often require large and diverse datasets, space-filling designs are particularly well-suited^23,24^.

Finally, the last category of experimental designs focuses on the qualification of specific regions within the input variable space—such as the Design Space or Normal Operating Region (NOR). Since it is impractical to validate every possible point within these regions, a common strategy involves using full or fractional factorial designs to evaluate only the corner points of a hypercubic region targeted for qualification. If the output specifications are satisfied at all—or nearly all—of these points, the region can be considered qualified. If not, the newly collected data can be leveraged to refine the predictive models and define an updated operating region for subsequent qualification.

Once the experimental design has been selected, the most critical and sensitive step is to execute the experiments as accurately and consistently as outlined in the initial plan. In drug development, these experiments are typically conducted on cell cultures (in vitro), laboratory animals (in vivo), or human subjects (in clinico). In contrast, for medical devices, where the mechanisms of action are generally well understood, it is often possible to use first-principles models to carry out simulated experiments (in silico), which can significantly accelerate the development process.

When the experimental design is chosen, the most delicate step is then to conduct the experiments as faithfully as possible to the initial plan. For drug development, these assays are carried out on cells (in vitro), on laboratory animals (in vivo), or on patients (in clinico). In contrast, for medical devices, since the mechanisms of action are known, first-principles models can be used to perform simulated experiments (in silico). In this case, it further accelerates the product development.

#### Statistical analysis of experimental data (STA)

As noted in the previous paragraph, the methods and statistical tools traditionally used to identify mathematical models—such as least squares estimators—are well established. However, with the growing influence of artificial intelligence, it is increasingly likely that new, data-driven techniques will be adopted at this stage to improve the predictive accuracy and robustness of the models.

Some machine learning techniques, such as random forests, are also highly effective for conducting global sensitivity analyses^25^. When it comes to identifying input regions that meet output specifications, response surface models are commonly applied. However, specification probability plots (SPP) offer a more robust alternative, as they explicitly incorporate modeling uncertainty into their predictions. These plots represent, across the entire experimental domain, the probability that the output specifications will be satisfied. As defined in Eq. (4), all regions where this probability exceeds a predefined acceptability threshold constitute the design space. Within this space, a subsequent sprint will focus on qualifying a hypercubic operating region.

#### SPP computation

The construction of specification probability plots relies on an estimation of the probability density function of *Y*. In most cases, we cannot obtain the exact and explicit form of the posterior distribution, and the best we can do is to get a sample from it by using numerical methods such as MCMC algorithms^26^ or the ABC method^27^. The R Project for Statistical Computing and two specific packages, rstanarm and rstantools, were used in this study to estimate the distribution of *Y*. These tools have the advantage of taking into consideration the estimation uncertainties of $\sigma$ and the control level of the input parameters. To build the specification probability plots, the experimental domain was decomposed according to a linear grid, where each range of input factors was divided into $(m-1)$ segments. For each of the $m^p$ points on the computation grid, the probability $\text {Prob}[Y \in \Lambda ]$ was estimated from $N = 1000$ samples. Further details on the calculation procedure are provided in^28,29^.

#### SPP visualization

When the number of input variables $p>2$, visualizing the specification probability plots becomes challenging. To overcome this difficulty, we use a matrix in which each element is a SPP(i,j), i.e. a map representing the calculated values of the probability $\text {Prob}[Y \in \Lambda ]$ on a grid of values for the two input variables $u_i$ and $u_j$ distributed along the two axes. In SPP(i,j), the other input factors $u_k$ with $k\ne \{i;j\}$ are not fixed but are randomly drawn according to a uniform distribution limited to their experimental domain $[u_{k,min}; u_{k,max}]$.

### Tests of qualification

As illustrated in Fig. 1, once the final step of the investigation cycle—statistical analysis (STA)—is completed, four possible outcomes may arise:

1. The analysis yields a positive and sufficiently accurate answer to the sprint question, enabling the innovation process to advance to the next sprint ($S\rightarrow S+1$ in Fig. 2).
2. The analysis provides only a partial or inconclusive answer with limited accuracy, prompting either a repeat of the current sprint ($S.k\rightarrow S.k+1$) or a return to a previous sprint ($S\rightarrow S-i$, where $i\ge 1$) for further refinement.
3. The analysis delivers a negative outcome, suggesting the original objective is not viable. In this case, the project may pivot to explore alternative applications or use cases for the product.
4. The findings indicate that the product cannot meet the required performance specifications, resulting in a decision to terminate the development at this stage. This scenario aligns with the “fail fast” strategy commonly advocated in pharmaceutical innovation^30^.

When all the critical development questions grouped within a backlog corresponding to a given Technology Readiness Level (TRL) have been satisfactorily addressed, the process can advance to the questions associated with the next TRL ($T\rightarrow T+1$). Figure 2 illustrates how the QbD framework supports iterative innovation through sequential sprints.

### Agile QbD implementation

To apply this innovation procedure grounded in the agile QbD model, we used easyQBD 1.1 (https://qbd.cybernano.eu/), a cloud-based SaaS platform developed through two European projects: TBMED (supported by the European Union research and innovation Horizon 2020, grant no. 814439) for the development of high-risk medical devices and EXPERT (supported by the European Union research and innovation Horizon 2020, grant no. 825828) for the development of mRNA-based drugs against cancer. The algorithms used by easyQBD to design experiments and analyze data were developed in the open-source R language.

### A study case

#### A new radiopharmaceutical for PET imaging

To diagnose prostate cancer in PET imaging, since few years, the main radiotracer used is the 68Ga-PSMA-11^32^. The synthesis of this radiopharmaceutical is easy and efficient (RCY $> 80\%$), fast (less than 30 min) and consumed some micrograms of radiolabeling precursor thanks to complexation step for radiolabeling but due to half-live of 68Ga, it expiration date is very short. Other radiopharmaceuticals to target Prostate-Specific Membrane Antigen (PSMA) have been developed, with 18Fluorine as radionuclide allowing to envisioned a commercialization of the product due to its longer half-live allowing to extend the expiration date compare to [$^{68}$Ga]Ga-PSMA-11. But the radiosynthesis is longer, the radiolabeling yield is lower than [$^{68}$Ga]Ga-PSMA-11 and involved a quantity of radiolabeling precursor of the order of few milligrams. Recently, new radiopharmaceutical like [$^{18}$F]AlF-PSMA-11 combining advantage of complexation radiochemistry with fluorine-18 radionuclide have been developed^33^. This case study examines how Agile QbD can be used in the preclinical phase to optimize the yield of the radiolabeling step for this type of radiopharmaceutical, as shown in Fig. SF1 (Supplementary File). Now, we specify the characteristics of the experiments carried out within the QbD sprints.

#### Experimental section

Reagents and general methods

Sodium chloride solution for injection, 0.9% (wt/vol) was purchased from B. Braun. Anhydrous AlCl$_3$, 99.999% (wt/wt), trace metals basis and sodium acetate, 99.99% (wt/wt) trace metals basis were purchased from Sigma Aldrich. Acetic acid glacial ($>99.85\%$) was supplied by Merck. Anhydrous absolute ethanol was purchased from Carlo Erba. PSMA-11 was purchased from MedKoo Biosciences (Morrisville, USA). Milli-Q water (18.2 M$\Omega \cdot$cm) was used for the aqueous solution preparation. Sep-Pak^®^ Accell Plus QMA Plus Light Cartridge (130 mg Sorbent per Cartridge, 37–55 $\upmu$m), Oasis HLB was purchased from Waters. Buffer was prepared and controlled with Mettler Toledo pH-meter with InLab^®^Micro pH electrode. The activity measurements were carried out using the radioisotope dose calibrator (CRC^®^-25R, Capintec, Inc.). No-carrier-added fluoride-18 was produced via the $^{18}$O (p,n)$^{18}$F nuclear reaction on a PET Trace cyclotron (GE). The bombardment was performed at 10 $\upmu$A during 5 min to provide about 5 GBq of fluoride-18 delivered as a solution in $^{18}$O-enriched water (1.6 mL). Manual radiosynthesis were performed using ThermoMixer^®^ from Eppendorf. Automated radiosynthesis were carried out on an AllInOne^®^ synthesis module (Trasis, Ans, Belgium).

Quality control procedure

Radiosynthesis were monitored by radio-TLC using silica plate as stationary phase and H$_2$O/CH$_3$CN (40/60, v/v) as mobile phase. TLC plates were revealed using TLC-scanner mini-GITA^®^ (Elysia Raytest, Straubenhardt, Germany). Gina X software (Elysia Raytest, Angleur, Belgium) was used for operation of chromatograph acquisition and processing of data. Radiosynthesis were monitored by UHPLC using NEXERA LC40 XS (Shimadzu) system controlled by LabSolution software (Kyoto, Japan) equipped with a PhotoDiode Array Detector SPD-M40 (Shimadzu, Marne-la-Vallée, France) in serie with a radio HPLC detector (Herm LB500 with fLumo detector) from Berthold (Bad Wildbad, Germany). The system was equipped with an ACE^®^ Avantor excel2 superC18 column (50 x 2.1 mm, 2 $\upmu$m, 90) and separation was achieved using the following gradient: 5% B for 0.5 min, 5% to 40% B in 4.5 min (A: H$_2$O, 0.1% of TFA, B: CH$_3$CN, 0.1% TFA). The flow rate was 0.8 mL/min and the injection volume was 3–10 $\upmu$L. The peaks were detected with UV detection at 280 nm and radioactive detection.

**Fig. 3:**
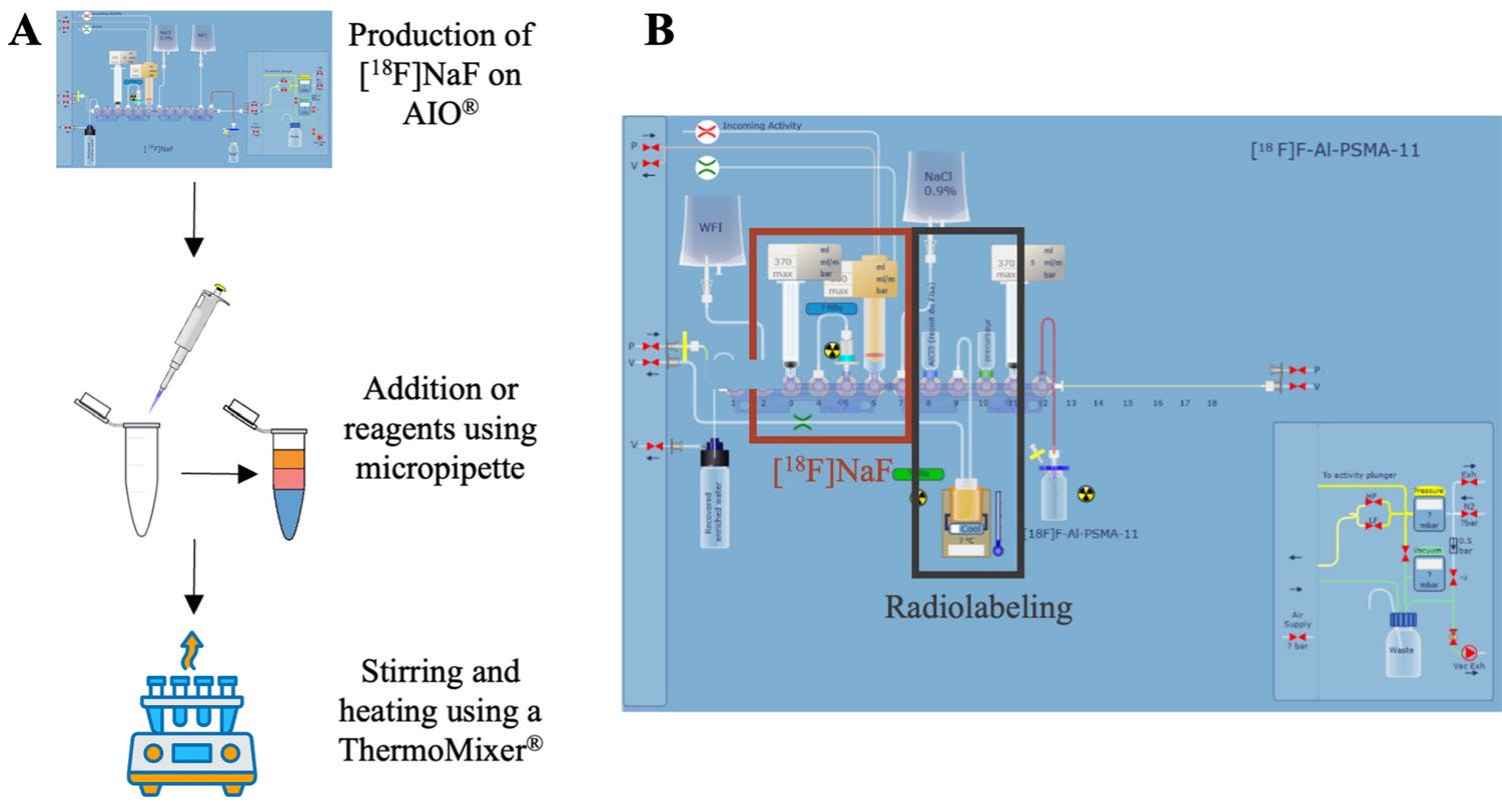
(**A**) Manual Al$^{18}$F-labeling of PSMA-11. (**B**) Layout of automated Al$^{18}$F-labeling of PSMA-11 on AIO^®^ (Trasis).

General method for the radiolabeling of [$^{18}$F]AlF-PSMA-11 in manual mode, (Fig. 3A)

[$^{18}$F]NaF was synthetized on AllInOne^®^ module following the protocol of Collet et al.^34^. Briefly, [$^{18}$F]NaF was eluted from a Sep-Pak^®^ Accell Plus QMA Plus Light Cartridge with NaCl 0.9% (3 mL) with RCY of 81±3% dc (n = 12). [$^{18}$F]NaF solution (500 $\upmu$L, 50 MBq) was added to a aluminum chloride solution at 2 mM in NaCl 0.9% (5–60 $\upmu$L, 0.5, 1 or 1.5 equiv. relative to amount of PSMA-11) in NaOAc buffer 0.1 M pH 4.5 (125 $\upmu$L) and EtOH (97–1780 $\upmu$L). The solution was stirred at RT for 5 min 300 rpm using a ThermoMixer^®^. A solution of PSMA-11 of 1 mg/mL in MilliQ water (20, 50 or 80 $\upmu$L, 1 equiv.) in NaOAc buffer 0.1 M pH 4.5 (125 $\upmu$L) and EtOH (97–1780 $\upmu$L) were added and the radiolabeling was performed at 35, 50 or $65^{\circ }$C for 15 min (300 rpm) (EtOH rate of 20, 50 or 80%). For RCC quantification, the crude mixture was collected in the final vial. Quality control procedure was applied to determine the RCP of the product by radio-UHPLC (Rt: 3.05 min) and by radio-TLC (FR: 0.8) using the conditions described above.

General method for the radiolabeling of [$^{18}$F]AlF-PSMA-11 on AllInOne^®^ (Trasis) (Fig. 3B)

The cassette description is presented Table 1. [$^{18}$F]Fluoride (500 MBq) delivered as a solution in $^{18}$O-enriched water (1.6 mL) was pumped into the plunger of the AIO^®^ module. [$^{18}$F]Fluoride was trapped on a Sep-Pak^®^ Accell Plus QMA Plus Light Cartridge and washed with water for injection (3 mL). [$^{18}$F]Fluoride was eluted in [$^{18}$F]NaF form thanks to NaCl 0.9% (0.5 mL) and placed into the reactor. The aluminum solution (5-60 $\upmu$L AlCl$_3$ 2 mM in NaCl 0.9% in NaOAc pH 4.5/EtOH at different proportions (1.5 mL)) (0.5, 1 or 1.5 equiv.) was transferred and Al$^{18}$F$^{2+}$ adduct was formed under nitrogen bubbling (150 mbar) for 5 min at RT. PSMA-11 solution (20, 50 or 80 $\upmu$L of PSMA-11, 1 mg/mL in H2O in NaOAc pH 4.5/EtOH at different proportions (1.5 mL)) was transferred in the reactor. The radiolabeling was performed at 35, 50 or $65^{\circ }$C for 15 min in the sealed reactor and then was cooled to $30^{\circ }$C. For RCC quantification, the crude mixture was collected in the final vial. Quality control procedure was applied to determine the RCP of the product by radio-UHPLC (Rt: 3.05 min) and by radio-TLC (FR: 0.8) using the conditions described above.

**Table 1:** Position on manifold of material and reagents for automated radiosynthesis of [$^{18}$F]F-Al-PSMA-11.

Manifold position	Materials or reagents
1 Horizontal	Silicone tubing to pressure inlet
1 Vertical	Silicone tubing to [$^{18}$O]H$_2$O recovery vial
2	Water for injection (WFI) bag
3	Syringe of 10 mL (S1)
4	Silicone tubing to QMA cartridge at position 5
5	Sep-Pak^®^ light Accell plus QMA cartridge
6	[$^{18}$O]H$_2$O/[$^{18}$F]F inlet reservoir (S2) (plunger)
7	NaCl 0.9% (wt/vol) bag
8	Aluminum solution vial of 4 mL
9	Silicone tubing to reactor
10	Precursor solution vial of 4 mL
11	Syringe of 10 mL (S2)
12 Vertical	Silicone tubing to final vial
12 Horizontal	Silicone tubing to exhaust

### Application of agile QbD to the preclinical development of a new radiopharmaceutical

The Agile QbD approach was applied to the development of a novel radiopharmaceutical through six consecutive sprints. The study placed particular emphasis on the radiolabeling step, which represented the most significant challenge in achieving the desired performance—especially with respect to the radiochemical conversion rate (RCC). The first three sprints focused on the manual synthesis of the compound, while the subsequent sprints addressed its automated production. At the outset, the product was in the concept phase (TRL 2) and progressed to TRL 4 by the end of the project. This innovation journey was guided by five key development questions, defined by the project team and organized into a development backlog, as detailed in Table 2.

**Table 2:** Backlog of questions addressed during the development of the new radio-conjuguate between TRL 2 and 4.

Sprint	Question
Q.S1	As a developer, what are the critical input variables influencing the radiochemical conversion rate (RCC) during the manual synthesis ?
Q.S2	As a developer, what would be the range of values for the critical input variables that are compatible with the RCC specifications ?
Q.S3	As a developer, can the design space identified in the previous sprint be qualified to validate the initial proof of concept ?
Q.S4	As a developer, what happens to the design space when the synthesis is automated ?
Q.S5	As a developer, can the new operating region be qualified/validated with automated production ?

Sprint S1.1

To address the first development question, an initial sprint was carried out to identify the most critical input variables influencing the target output: the radiochemical conversion rate (RCC). The list of the input variables is given in Table 3. After establishing the first version of the Target Product Profile (TPP), a factor screening model—as defined in Eq. (1)—was applied. The variable *u*8 (temperature), already recognized as a critical factor, was excluded from the screening analysis. Model coefficients were estimated based on a dataset obtained from thirty pilot experiments, following the detection and elimination of highly correlated input variables to ensure model robustness. This dataset is given in Table SF1 of the Supplementary File whereas the correlation matrix is shown in Fig. SF2.

**Table 3:** Initial list of input ($u_i$) and output (*Y*) variables. Inputs are composed of material attributes and process parameters.

Not.	Definition	Type	L1 (or min)	L2 (or max)
$u_1$	Volume AlCl$_3$ 2 mM ($\upmu L$)	Quantitative	8	37.5
$u_2$	Equivalent number AlCl$_3$/PSMA	Quantitative	0.4	2.5
$u_3$	Precursor quantity ($\upmu g$)	Quantitative	20	85
$u_4$	Precursor concentration ($\upmu g /mL$)	Quantitative	5.8	28.9
$u_5$	Ethanol (%)	Quantitative	23.1	56.2
$u_6$	Volume total ($\upmu L$)	Quantitative	2045	4320
$u_7$	Labeling duration (min)	Quantitative	10	15
$u_8$	Temperature ($^{\circ }$C)	Quantitative	35	65
*Y*	Radiochemical conversion rate ($\%$)	Quantitative	$RCC>70\%$	$100\%$

Sprint S2.1

In the second sprint, a quadratic response surface model was employed to characterize the impact of the remaining four input variables on the target output. To generate the estimation dataset, a Hartley composite design was conducted in triplicate. Given that the output variable is strictly positive and constrained between 0% and 100%, a logarithmic transformation was applied prior to estimating the model coefficients. The transformed output variable, denoted as *Z*, is defined as follows:

$$Z=\log \left( \frac{Y/100}{1-Y/100}\right) ,$$

where *Y* represents the radiochemical conversion rate. To retain only the most relevant terms in the model, a stepwise regression approach was applied. The resulting model was then used to generate response surface plots of the predicted output values, enabling the identification of an operating region that meets the initial performance requirements for *Y*.

Sprint S3.1

In the third sprint, a four-dimensional hypercube was extracted from the previously generated response surfaces to serve as the candidate operating region for qualification. The strategy involved testing the corner points of this hypercube to assess the robustness of the internal region. For a four-dimensional space, this would normally require evaluating 16 distinct experimental conditions. To minimize the associated experimental burden, a fractional factorial design $2^{4-1}$ was used, allowing the assessment of 8 selected corners out of the 16. The details of this design are provided in the Supplementary file, Table SF5.

Sprint S4.1

This sprint mirrors Sprint S2.1, but was conducted during the automated synthesis of the radiopharmaceutical. The experimental design followed the same structure, utilizing a Hartley composite design. To optimize time and reduce costs, each experimental condition was performed once, except for the central point, which was replicated five times. The data analysis followed the same methodology; however, to better account for modeling uncertainty in identifying a suitable operating region, a design space was calculated rather than relying solely on response surface plots.

Sprints S5.1 and S5.2

As in Sprint S3.1, the objective at this stage was to qualify an operating region that satisfies the specifications of the output variable. To achieve this, two consecutive sprints were conducted, each employing a different experimental design. The corresponding designs, consisted of eight experiments each, are detailed in the Supplementary file.

## Results

**Fig. 4:**
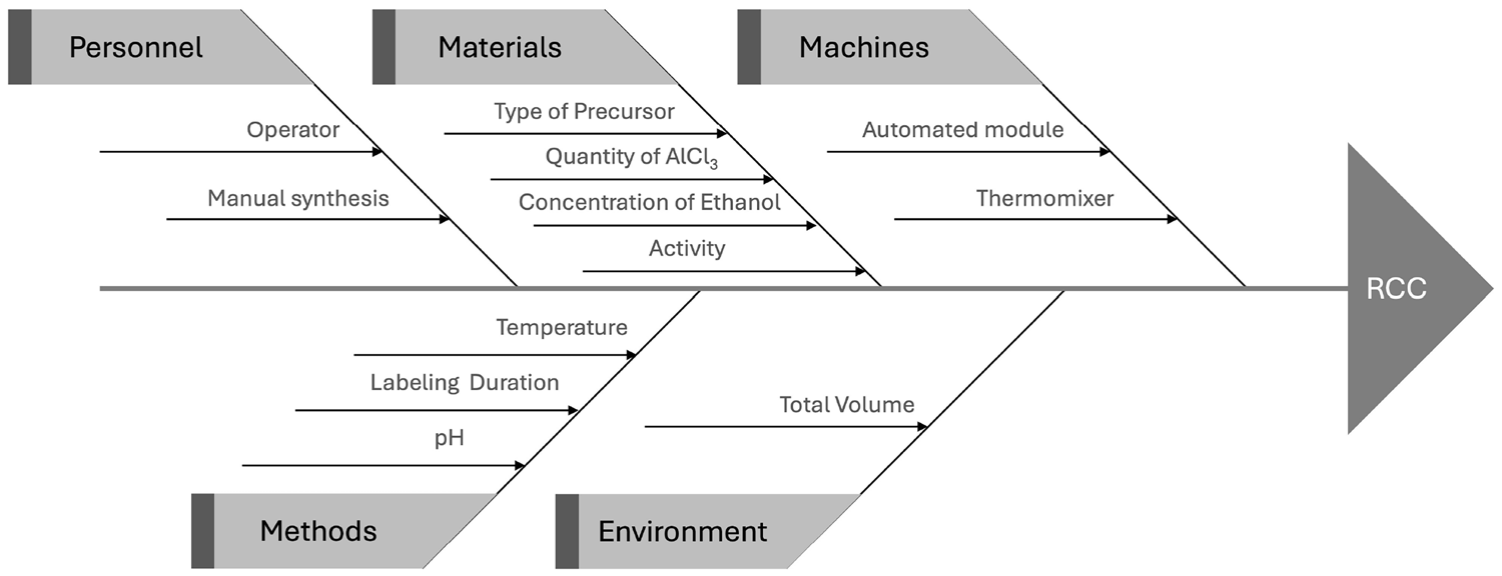
Cause-effect diagram of the radiolabeling parameters on the radiochemical conversion rate (RCC).

The initial Target Product Profile (TPP) for the new radiopharmaceutical is outlined in Table 4. Following a breakdown of the synthesis process, the radiolabeling step was identified as the most critical stage, with particular emphasis placed on a key Critical Quality Attribute (CQA): the radiochemical conversion rate (RCC), which was targeted to exceed 70%. As part of the Input-Output Modeling (IOM) phase of the first sprint *S*1, a cause-and-effect (Ishikawa) diagram was developed (Fig. 4). This diagram compiles all potential sources of RCC variability, as identified by expert input, serving as the foundation for further investigation and model development.

**Table 4:** Summary of the target product profile at the beginning of the innovation process.

Feature	Information
Name	[$^{18}$F]F-Al-PSMA-11
Short Description	$^{18}$F-radiopharmaceutical composed of a PSMA receptor targeting agent and a chelator for Al-$^{18}$F
Indication	Diagnosis of prostate cancer by PET imaging
Composition	[$^{18}$F]F-Al-PSMA-11 formulated solution containing 10% EtOH
Mechanism of action	Specific recognition of PSMA receptor
Route of administration	Intravenous
Dosage	1–2 MBq / kg
Market differentiation	Better image contrast for earlier detection
Packaging	Sterile sealed glass vial
Predicate	$^{18}$F-analog to [$^{68}$Ga]Ga-PSMA-11

QbD sprint S1.1

**Fig. 5:**
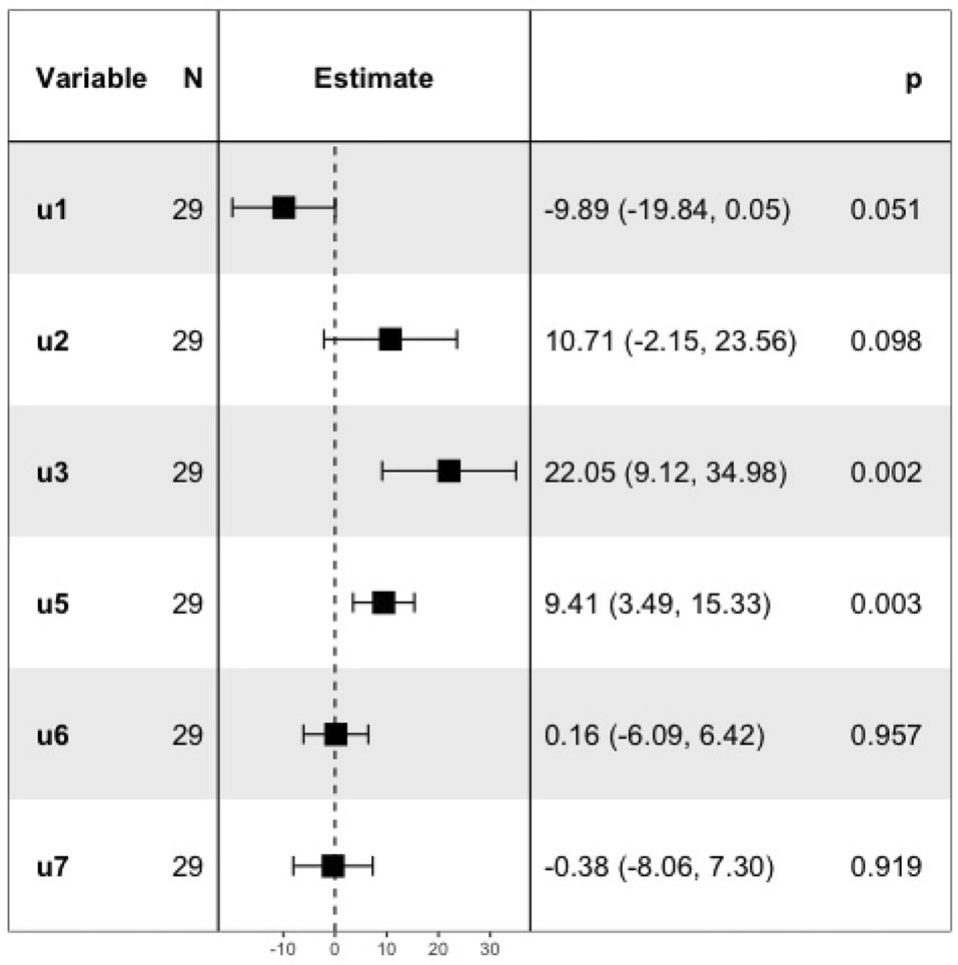
Forest plot of the screening model, sprint *S*1.

Figure 5 presents a Forest plot that displays and compares the additive effects estimated for each of the six input variables tested in the screening study. The last column of this diagram is composed of the mean value, the 95% confidence interval and the p-value for each input effect. It is important to recall that variables $u_4$ and $u_8$ were excluded beforehand: $u_4$ due to its strong correlation with $u_3$ ($\rho \approx 0.9$), as emphasized by a correlation matrix given in the Supplementary File, Fig. SF2, and $u_8$ because it was already known to be a critical factor. Among the six tested variables, three—$u_2,u_3,$ and $u_5$—were identified as critical based on their positions and confidence intervals in the Forest plot (Fig. 5). The most influential factor was $u_3$, representing the precursor quantity, with a mean additive effect of approximately $b_3\approx 30.85$. Its large magnitude and tight 95% confidence interval suggest a highly significant contribution. Both $u_1$ and $u_2$ are related to AlCl$_3$: $u_1$ corresponds to its absolute quantity, while $u_2$ reflects the molar equivalent relative to the precursor. Their estimated effects are similar in absolute terms, though the effect of $u_2$ carries slightly more uncertainty. Given that molar equivalents are more commonly used in the literature, $u_2$ was retained over $u_1$. The third selected input variable, $u_5$ (ethanol concentration), also demonstrated a significant positive effect on the output. In contrast, $u_6$ and $u_7$ were determined to be non-critical, as indicated by their small mean effects and large p-values. With these insights, the objective of this sprint has been successfully achieved, enabling the project to advance to the next investigation question and subsequent sprint.

QbD sprint S2.1

In this second sprint, the objectives were twofold: (1) to deepen the understanding of the cause-and-effect relationships between the four selected input variables ($u_2, u_3,u_5,u_8$) and the radiochemical conversion rate (*Y*), and (2) to identify an optimal operating region within the four-dimensional input space—specifically, a subregion where each operating point is likely to meet the output specification. Given that *Y* was assessed using two distinct analytical protocols—UPLC and CCM—the study considered two output variables: $Y_1$ corresponding to UPLC measurements, and $Y_2$ to those from CCM. The experimental data for both outputs, obtained through the Hartley composite design, are provided in the Supplementary file, Table SF2. A slight systematic bias was observed between the $Y_1$ and $Y_2$ measurements, indicating a potential need for a future Analytical QbD study to better harmonize the two methods^35^. The dataset was then used to estimate the coefficients of the corresponding investigation models. Following a logarithmic transformation of the measured responses and the application of stepwise regression, the final models for the transformed responses, $Z_1$ and $Z_2$, are defined as follows:

5 $$\begin{aligned} Z_1&= b_0+ b_2\,u_{2} + b_3\,u_{3} + b_5\,u_{5} + b_8\,u_{8} + b_{55}\,u_{5}^2 + b_{25}\,u_{2}u_{5} + b_{38}\,u_{3}u_{8} +b_{58}\,u_{5}u_{8}+E_1 \end{aligned}$$

6 $$\begin{aligned} Z_2&= b_0+ b_2\,u_{2} + b_3\,u_{3} + b_5\,u_{5} + b_8\,u_{8} + b_{33}\,u_{3}^2 + b_{55}\,u_{5}^2 + b_{28}\,u_{2}u_{8} +b_{58}\,u_{5}u_{8}+E_2, \end{aligned}$$

with $$E_1\sim \mathcal {N}(0,\sigma _1^2)$$ and $$E_1\sim \mathcal {N}(0,\sigma _2^2)$$.

**Fig. 6:**
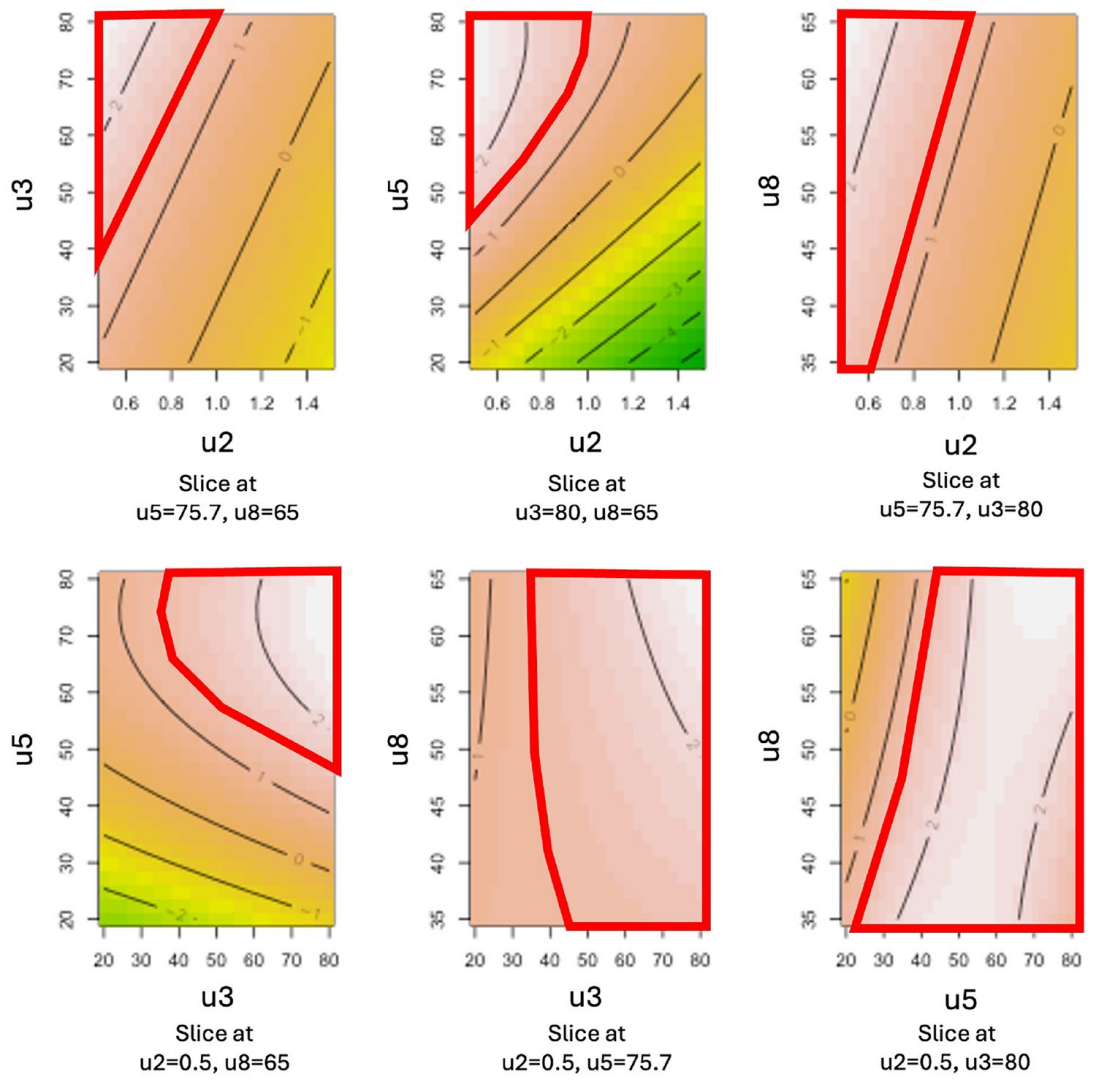
Matrix of predicted response surfaces for $Z_1$, sprint S2.

**Fig. 7:**
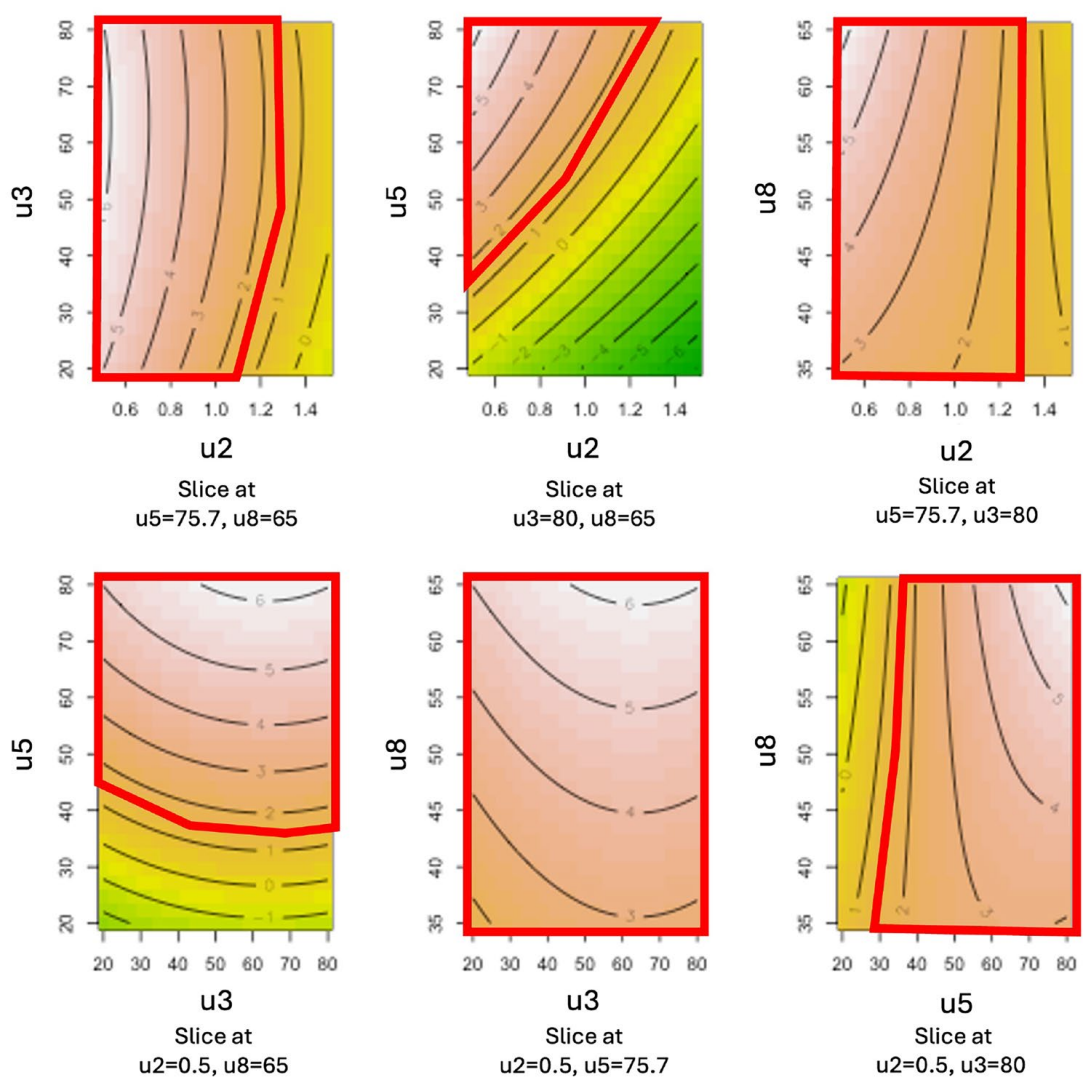
Matrix of predicted response surfaces for $Z_2$, sprint S2.

The coefficients of determination for the two models are approximately $R_1^2\approx 0.814$ and $R_2^2\approx 0.793$, indicating a good fit in both cases. The corresponding estimated model coefficients are provided in the Supplementary file, Tables SF3 and SF4. The predicted response surfaces generated from these models are illustrated in Figs. 6 and 7. Meeting the specification $Y>70\%$ is equivalent to satisfying the transformed condition $Z>0.85$. The regions of the input space where this criterion is met are highlighted in red on the response surfaces. The substantial size of these regions confirms that the RCC performance target is achievable and further delineates the optimal operating zone on which to concentrate efforts in subsequent development phases.

QbD sprint S3.1

This new sprint focuses on qualifying an operating region derived from the response surfaces generated in the previous sprint. The strategy involves testing the corner points of a four-dimensional hypercube, positioned within the red-highlighted regions shown in Figs. 6 and 7. In a four-dimensional space, this would normally require evaluating 16 experimental conditions. To limit the associated experimental cost, a fractional factorial design $2^{4-1}$ was employed, selecting 8 out of the 16 corners for testing. The results of these experiments are provided in the Supplementary file, Table SF5. For both analytical methods used, all eight test points exceeded the specification threshold, confirming the suitability of the selected region. This sprint successfully addresses the development question Q3, which aimed to demonstrate the validity of the operating region for the manual synthesis of the radiopharmaceutical. With this proof of concept established, the project progresses to Technology Readiness Level (TRL) 3.

QbD sprint S4.1

**Fig. 8:**
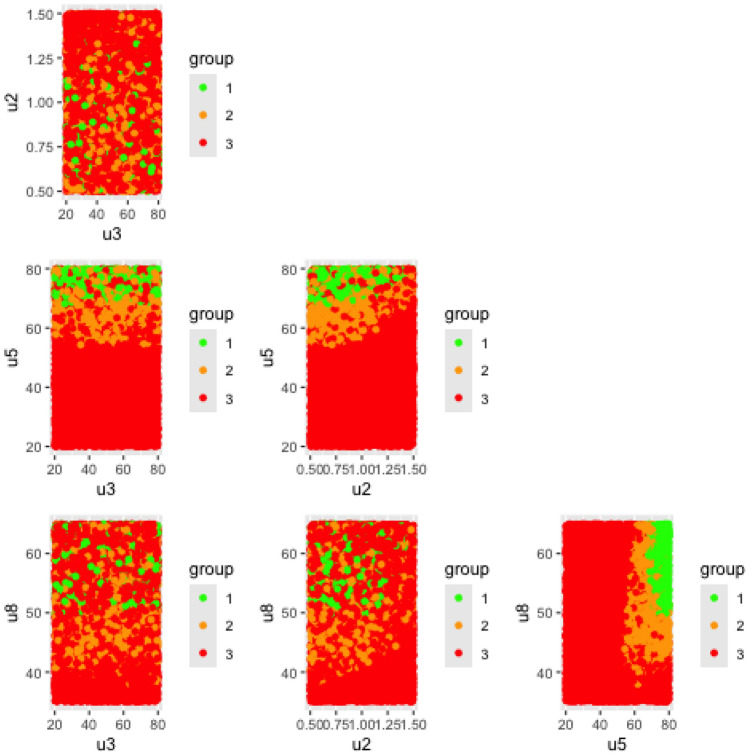
Specification probability plots (SPP) for $Z_1$, Sprint *S*4.1. Green area denotes the design space, i.e. the operating region in which , while the two other colors correspond to:  and .

**Fig. 9:**
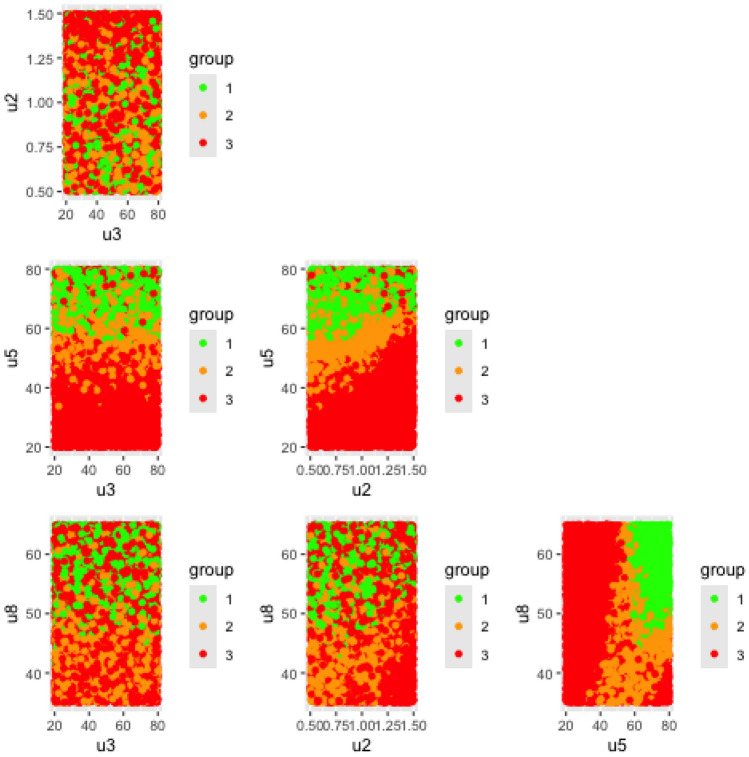
Specification probability plots (SPP) for $Z_2$, Sprint *S*4.1. Green area denotes the design space, i.e. the operating region in which , while the two other colors correspond to:  and .

This fourth sprint marks the beginning of a new development phase, during which the radiopharmaceutical synthesis is carried out using an automated production system, illustrated in Fig. 3. To evaluate whether the cause-and-effect relationships between the four previously identified critical input variables and the radiochemical conversion rate (RCC) remained consistent under the new production conditions, the same experimental design used in Sprint S2 was applied. The resulting dataset is given in Table SF6. Unlike Sprint S2, where response surface models were used, this sprint employed the construction of a design space to better account for modeling uncertainty. The resulting Specification Probability Plots, whose construction is described in section 2.2.4, are shown in Figs. 8 and 9 for the transformed responses $Z_1$ and $Z_2$ respectively. In both figures, a green region is observed in the SPP(u5, u8) diagram, at the bottom right of the matrix. It corresponds to the design space and its shape depends only on $u_5$ and $u_8$, thus stressing their status as critical process parameters. In this area, the specifications for $Z_1$ and $Z_2$ are met whatever values $u_2$ and $u_3$ may take, thus indicating these two input variables are less critical than $u_5$ and $u_8$. Estimates of the coefficients associated with the two models are given in Tables SF7 and SF8 for $Z_1$ and $Z_2$ respectively.

*QbD sprints S5.1 and S5.2*

**Fig. 10:**
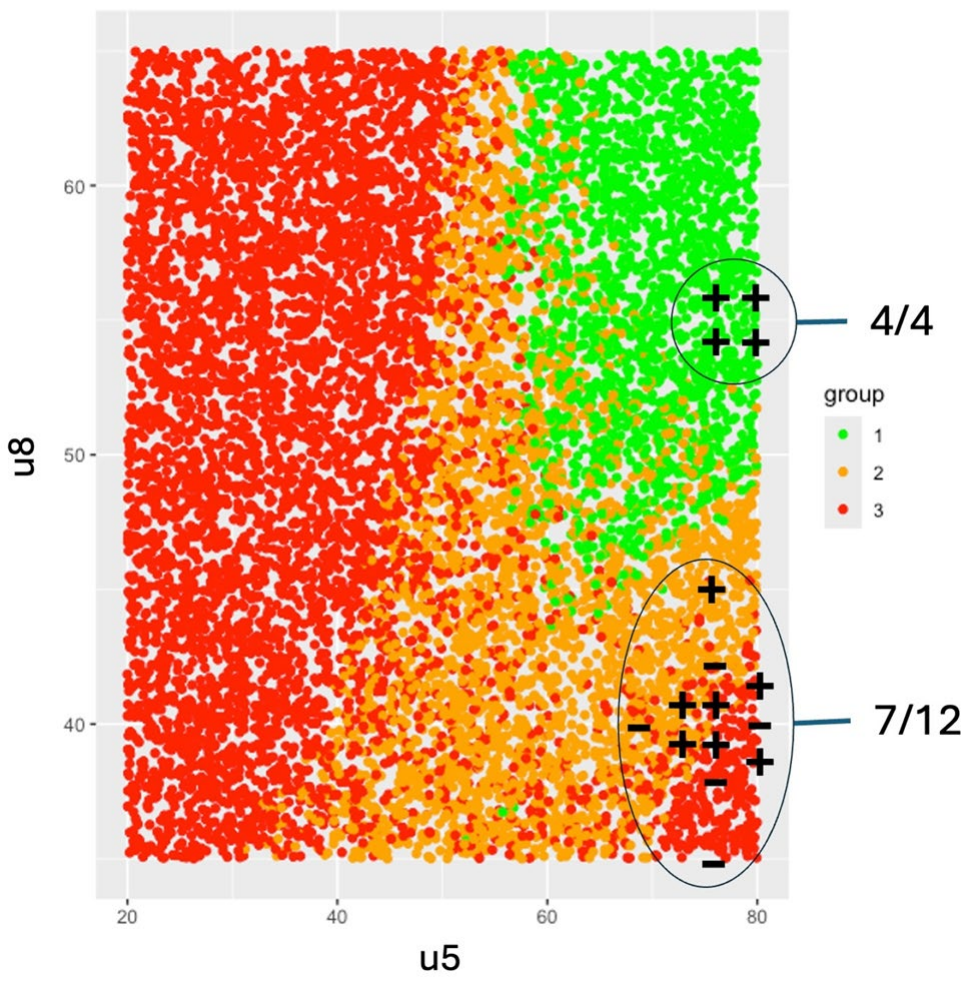
Qualification of the design space during automated production of a PET imaging radiopharmaceutical. Symbol $+$ denotes experiments whose results are in compliance with the RCC specification while − are negative tests, i.e. out of specification responses. Design space:  and out-of-specification regions:  and .

An additional sprint, S5.1, was conducted to qualify the design spaces previously identified (green regions in Figs. 8 and 9). A first experimental design, detailed in the Table SF9, was implemented. The results confirmed that all tested conditions within the green zone met the RCC specification. However, since most test points fell within the design space, the experiment did not allow for validation of whether the orange and red zones, predicted to be non-compliant, actually failed to meet the specifications. To address this gap, a second iteration of the sprint (S5.2) was carried out using a new experimental design, given in the Table SF10, this time focusing on points located outside the design space. The combined results of both qualification experiments are summarized in Fig. 10, which illustrates the design space (green area) for $Z_2$ as a function of $u_5$ and $u_8$, while $u_2$ and $u_3$ were allowed to vary freely within their experimental domains. Test outcomes are marked with ‘+’ and ‘–’ symbols, indicating whether the corresponding $Y_2$ values complied with the output specifications. As predicted, all four tests conducted within the design space met the required criteria. Outside the design space, only 7 out of 12 tests ($\approx 58\%$) met the specifications, which is also consistent with model predictions. As a result, for the next development phase, the values of $u_5$ and $u_8$ will be selected within the validated design space:

- Ethanol concentration: $$u_5\in [75;80]\%$$
- Temperature: $$u_8\in [55;60]^{\circ }C$$,

while $u_2$ and $u_3$ can be arbitrarily set within the following ranges:

- Equivalent number AlCl$_3$/precursor: $u_2\in [1;1.2]$
- Precursor quantity: $$u_3\in [75;80]\mu g$$.

**Fig. 11:**
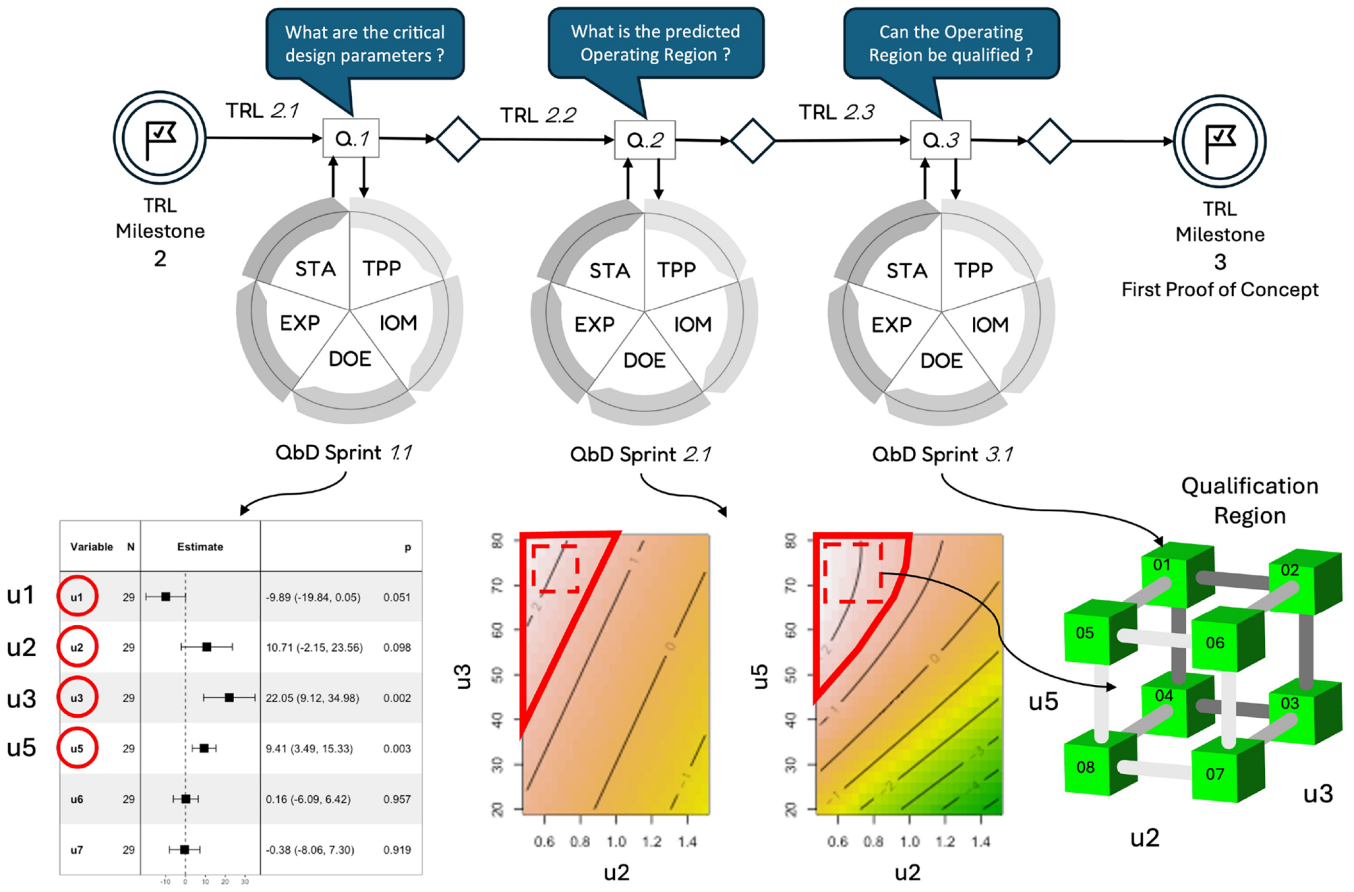
Application to the development of a PET imaging : from TRL 2 to 3.

**Fig. 12:**
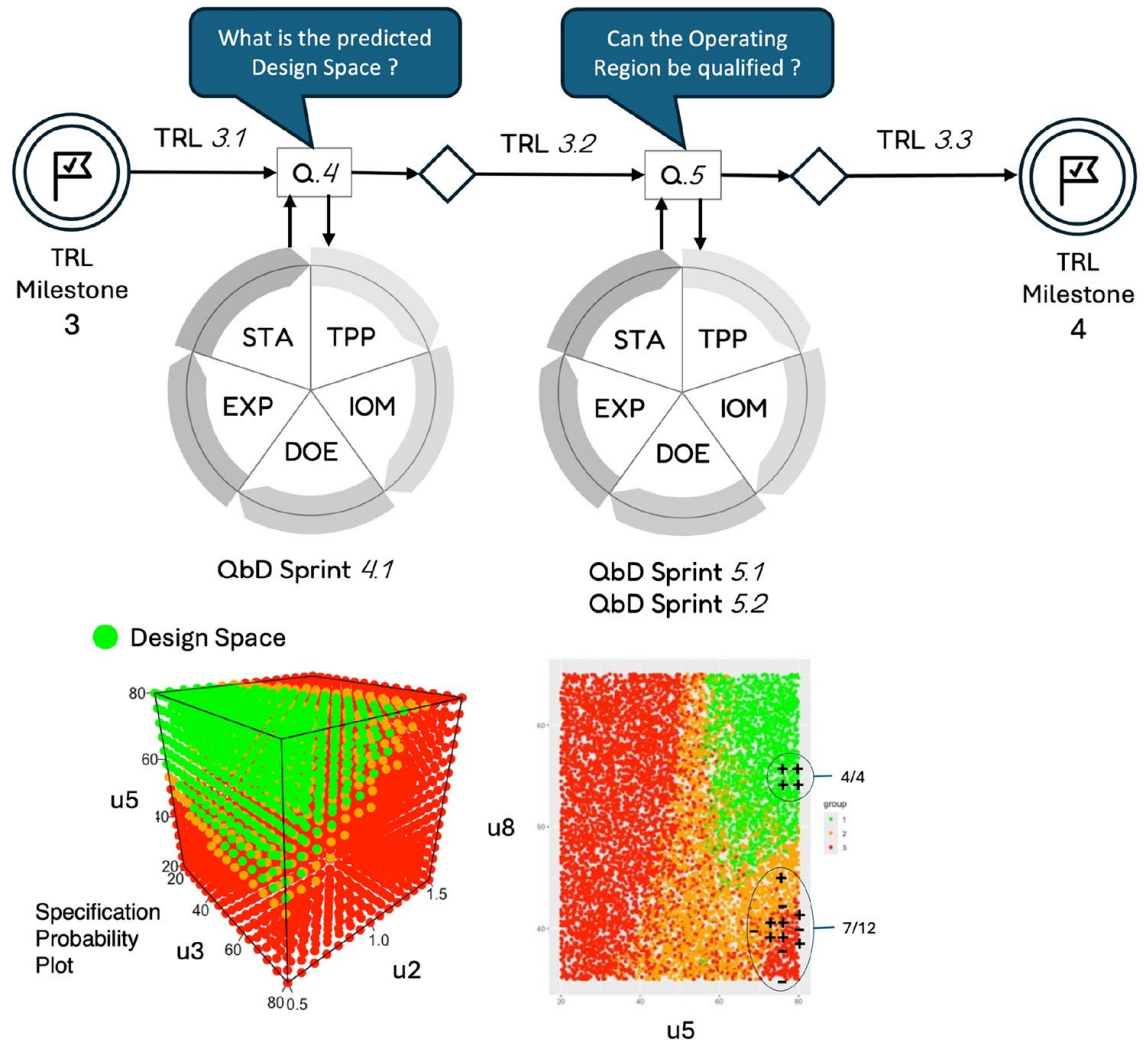
Application to the development of a PET imaging : from TRL 3 to 4.

## Discussion

This study introduces an agile organization of the Quality by Design (QbD) approach, that aims at making the methodology more systemic and more adaptable to the inherent uncertainties of drug development. Our ambition is also to broaden the scope of QbD to preclinical studies in order to obtain authorizations for the first clinical trials more rapidly. The core objective of this transformation is to provide a structured yet flexible framework that allows teams to respond swiftly and effectively to new information or unexpected challenges. The agile QbD model is built around the iteration or incrementation of short, focused studies, referred to as sprints, which are aligned with the Technology Readiness Level (TRL) scale. Each sprint is designed to address a specific priority development question, following a hypothetico-deductive investigation cycle to formulate and test hypotheses. A typical QbD sprint consists of five key steps:

- Developing and updating the target product profile (TPP)
- Identifying critical input and output variables
- Designing the experimental strategy
- Conducting the experiments
- Performing a statistical analysis of the collected data to answer the development question addressed by the sprint.

All critical questions defined by the development team are compiled into a backlog, which can be tackled sequentially. To support standardization and generalization, three question formats with specific syntaxes were also proposed. At the conclusion of each sprint, one of four decisions can be made:

- The knowledge about the product has increased, and we can move on to the next question and sprint
- Iterate the current or a previous sprint to reduce decision risk
- Pivot to redefine the product profile
- Terminate the development project.

These decisions are guided by the outcomes of statistical analyses that estimate the likelihood of meeting the required efficacy, safety, and quality specifications of the medicinal product under development.

Beyond its agile restructuring, our approach introduces several key enhancements to the traditional QbD framework. One major improvement is the introduction of a new component: the development backlog, inherited from the agile Scrum method. It contains all the priority development questions to be addressed. This addition brings a short-term, actionable perspective to complement the TPP’s long-term vision of the final product, thereby enhancing the overall management and strategic alignment of QbD studies. Although the TPP is inherently intended to be a dynamic document, existing QbD methodologies do not specify when or how it should be updated. In contrast, the agile QbD model provides a clear mechanism for updating the TPP at the end of each sprint, using the insights gained from experimental results. Furthermore, while the product and its manufacturing process are inherently interlinked, conventional TPPs typically omit any reference to the process itself. To address this gap, we propose adding a dedicated section for process mapping, ensuring that process innovation is considered from the outset. In addition, we aimed to formalize and generalize the Input-Output Modeling (IOM) step by introducing a mathematical hypothesis framework tailored to the three generic types of development questions defined in the previous step. This structure provides a clear rationale for selecting an appropriate experimental design method in the third step of each QbD sprint, promoting methodological consistency and scientific rigor throughout the development cycle.

In contrast to the traditional QbD approach—which typically requires a full decomposition of the manufacturing process before being applied to each individual unit—the agile QbD model centers around a set of key development questions defined by the project team. As a result, it is no longer necessary to have a complete understanding of the final production system or to wait until TRL levels 6–7, i.e. phase II clinical trials, to begin applying the methodology. In our case study, the agile QbD framework was successfully implemented at lower TRL levels (2–4), allowing us to leverage its benefits from the early stages of development. The application focused on the development of a new radiopharmaceutical for PET imaging, conducted over six consecutive sprints presented in Figs. 11 and 12. This iterative process enabled the project to progress from an initial product concept (TRL 2) to a functional prototype manufactured using an automated production system (TRL 4). Our findings demonstrate that QbD, when applied through an agile framework, is well suited to early-stage innovation and can be effectively integrated at low TRLs (2–3). In this study, each sprint lasted approximately two weeks, primarily dictated by the time needed to carry out experiments. For in vivo studies, this duration may increase by a factor of two or three due to additional complexity.

This agile restructuring of the QbD approach offers a key advantage: it enables the seamless integration of new objectives by simply adding additional sprints—without disrupting the overall organization of the project. This flexibility makes it particularly well-suited to accommodate the inherent uncertainties of innovation-driven development. By breaking QbD into discrete, manageable sprints, the agile model enhances the method’s responsiveness, allowing teams to address short-term priorities effectively while still maintaining a clear long-term vision of the final product as defined in the Target Product Profile (TPP). As outlined earlier, the quantitative treatment of each development question—within steps 2 (Input-Output Modeling), 3 (Design of Experiments), and 5 (Statistical Analysis)—provides measurable, verifiable outcomes, particularly useful in risk assessment, such as evaluating the probability of failing to meet product specifications. Importantly, this structural transformation of the QbD paradigm remains fully aligned with the regulatory framework set forth by ICH Q8^36^, as each sprint continues to adhere to its core principles. In summary, we believe that this clarified and modular organization of QbD significantly enhances its accessibility, comprehension, and practical application across development teams.

While this study underscores the flexibility and ease of implementation of the agile QbD approach, it also acknowledges several inherent limitations. By nature, the efficiency gains promised by QbD stem from its use of predictive mathematical models. However, the reliability of these models depends on access to high-quality, representative data and on the ability to carry out well-designed experiments—conditions that are not always achievable in sufficient quantity or variety. As a result, the experimental phase of a QbD sprint can quickly become resource-intensive, both in terms of time and cost. When available, process simulators offer a valuable alternative by enabling digital (in silico) experiments, which can partially offset these constraints by reducing the reliance on physical testing. Another limitation lies in the use of black-box models—commonly employed in statistical analyses and for constructing design spaces. While effective in many cases, these models may lack accuracy when applied to highly nonlinear processes. Their approximations are generally acceptable within narrow, well-defined operating regions, but their reliability diminishes outside those bounds. To overcome this, recent advances in machine learning and deep learning, including the use of artificial neural networks and random forests, provide promising alternatives for modeling complex behaviors. In parallel, the integration of physics- and chemistry-based theoretical models offers additional opportunities to improve prediction accuracy and model interpretability.

The analysis of a real-world case also revealed an additional limitation: in practice, it is often necessary to conduct multiple experimental designs in sequence. However, the associated statistical analyses are frequently carried out in isolation, without incorporating insights gained from previous experiments. This fragmented approach is inconsistent with the core principles of QbD, which emphasize cumulative learning and knowledge building from one sprint to the next. To address this issue, Bayesian learning methods offer a promising solution, as they enable the progressive integration of prior knowledge into current analyses, enhancing both model accuracy and decision-making^29^. A compelling direction for future research involves the development and implementation of advanced tools, such as Bayesian Deep Learning models, which combine the strengths of probabilistic reasoning and deep neural networks^37–39^.

## Conclusion

In conclusion, the methodology and findings presented in this study offer a fresh perspective on the application of Quality by Design (QbD) to improve knowledge management during the development of innovative medicinal products. The proposed approach supports the systematic capture of knowledge through a scientific framework that leverages machine learning tools to enhance the extraction of meaningful insights from experimental data. This method is fully aligned with a continuous learning process, structured according to the Technology Readiness Level (TRL) scale. Throughout the sprint-based workflow, technical and methodological knowledge is naturally shared among team members via collaborative work sessions. The outcomes of each sprint are consolidated into four structured reports, which can be stored in a centralized database to support efficient knowledge retention and transfer. We believe this agile QbD framework holds both methodological and practical value for strengthening innovation management practices from the earliest stages of preclinical development.

## Supplementary Information

Supplementary Information.

## Data Availability

Data is provided within the manuscript or supplementary information files

## References

[1] Thesing, T., Feldmann, C. & Burchardt, M. Agile versus waterfall project management: Decision model for selecting the appropriate approach to a project. *Proc. Comput. Sci.* **181**, 746–756 (2021).

[2] Royce, W.W. Managing the Development of Large Software Systems (1970) (2021)

[3] U.S Food and Drug Administration: Pharmaceutical cGMPs for the 21st Century - A Risk-Based Approach. Final Report (2004)

[4] Hubert, C. et al. Improvement of a stability-indicating method by quality-by-design versus quality-by-testing: A case of a learning process. *J. Pharmaceut. Biomed. Anal.* **88**, 401–409 (2014). 10.1016/j.jpba.2013.09.026 24176744

[5] Lambert, E. & Janjic, J. M. Quality by design approach identifies critical parameters driving oxygen delivery performance in vitro for perfluorocarbon based artificial oxygen carriers. *Sci. Rep.* **11**(1), 5569 (2021). 33692373 10.1038/s41598-021-84076-1 PMC7946885

[6] Ajayi, T. O., Poka, M. S. & Witika, B. A. Formulation and optimisation of bedaquiline nanoemulsions for the potential treatment of multi drug resistant tuberculosis in paediatrics using quality by design. *Sci. Rep.* **14**(1), 31891 (2024). 39738619 10.1038/s41598-024-83408-1 PMC11686176

[7] Saleem, M. T., Shoaib, M. H., Yousuf, R. I. & Siddiqui, F. RSM and AI based machine learning for quality by design development of rivaroxaban push-pull osmotic tablets and its PBPK modeling. *Sci. Rep.* **15**(1), 7922 (2025). 40050302 10.1038/s41598-025-91601-z PMC11885842

[8] Kim, M. K. et al. Analytical quality by design methodology for botanical raw material analysis: A case study of flavonoids in Genkwa Flos. *Sci. Rep.* **11**(1), 11936 (2021). 34099770 10.1038/s41598-021-91341-w PMC8185112

[9] Alqahtani, A., Alqahtani, T., Al Fatease, A. & Tolba, E. H. A quality by design HPLC method for cephalosporin analysis in pharmaceuticals and water samples with environmental impact assessment. *Sci. Rep.* **15**(1), 33 (2025). 39747994 10.1038/s41598-024-84647-y PMC11697007

[10] Gupta, A., Rachana, S., Moorkoth, S. & Dhas, N. Quality by design based ecofriendly HPLC analytical method for simultaneous quantification of erastin and lenalidomide in mesoporous silica nanoparticles. *Sci. Rep.* **15**(1), 8873 (2025). 40087405 10.1038/s41598-025-93331-8 PMC11909239

[11] Schwaber, K. & Beedle, M. *Agile Software Development with Scrum* (Prentice Hall PTR, 2001).

[12] Guthery, F. S. Deductive and inductive methods of accumulating reliable knowledge in wildlife science. *J. Wildl. Manag.* **71**(1), 222–225 (2007).

[13] Breder, C. D., Du, W. & Tyndall, A. What’s the regulatory value of a target product profile?. *Trends BiotechnoL.* **35**(7), 576–579 (2017). 28391988 10.1016/j.tibtech.2017.02.011

[14] Elder, D., & Teasdale, A. ICH Q9 Quality Risk Management. ICH Quality Guidelines: An Implementation Guide. 579–610 (Wiley, 2017)

[15] Charoo, N. A., Shamsher, A. A., Zidan, A. S. & Rahman, Z. Quality by design approach for formulation development: A case study of dispersible tablets. *Int. J. Pharmaceut.* **423**(2), 167–178 (2012). 10.1016/j.ijpharm.2011.12.024 22209997

[16] Testas, M. et al. An industrial case study: QbD to accelerate time-to-market of a drug product. *AAPS Open* **7**(1), 1–13 (2021).

[17] Waghule, T. et al. Quality by design (QbD) in the formulation and optimization of liquid crystalline nanoparticles (LCNPs): A risk based industrial approach. *Biomed. Pharmacother.* **141**, 111940 (2021). 34328089 10.1016/j.biopha.2021.111940

[18] Martinez-Marquez, D., Mirnajafizadeh, A., Carty, C. P. & Stewart, R. A. Application of quality by design for 3D printed bone prostheses and scaffolds. *PloS one* **13**(4), 0195291–0195291 (2018). 10.1371/journal.pone.0195291 PMC5896968 29649231

[19] Saltelli, A., Ratto, M., Andres, T., Campolongo, F., Cariboni, J., Gatelli, D., Saisana, M., & Tarantola, S. Global Sensitivity Analysis - The Primer. (Wiley, 2008)

[20] Wasserstein, R.L., & Lazar, N.A. The ASA Statement on p-Values: Context, Process, and Purpose. (Taylor & Francis , 2016)

[21] Lewis, G. A., Mathieu, D. & Phan-Tan-Luu, R. *Pharmaceutical Experimental Design* (Marcel Dekker, 2005).

[22] Box, G. E. P. & Draper, N. R. *Empirical Model-Building and Response Surfaces* (Wiley, 1987).

[23] Pronzato, L. & Müller, W. G. Design of computer experiments: Space filling and beyond. *Stat. Comput.* **22**(3), 681–701 (2012).

[24] Santner, T. J. et al. *Space-Filling Designs for Computer Experiments* (Springer, 2018).

[25] Antoniadis, A., Lambert-Lacroix, S. & Poggi, J.-M. Random forests for global sensitivity analysis: A selective review. *Reliabil. Eng. Syst. Saf.* **206**, 107312 (2021).

[26] Blum, M.G. Regression approaches for approximate Bayesian computation. arXiv preprint arXiv:1707.01254 (2017)

[27] Csilléry, K., Blum, M. G., Gaggiotti, O. E. & François, O. Approximate Bayesian computation (ABC) in practice. *Trends Ecol. Evolut.* **25**(7), 410–418 (2010). 10.1016/j.tree.2010.04.001 20488578

[28] Rozet, E., Lebrun, P., Hubert, P., Debrus, B. & Boulanger, B. Design spaces for analytical methods. *TrAC Trends Anal. Chem.* **42**, 157–167. 10.1016/j.trac.2012.09.007 (2013).

[29] Bastogne, T., Hassler, L., Bruniaux, J., Thomassin, M., Gidrol, X., Sulpice, E., & Navarro, F.P. A Bayesian implementation of quality-by-design for the development of cationic nano-lipid for siRNA transfection. *IEEE Trans. NanoBiosci.* **22**(3) (2023) 10.1109/TNB.2022.3213412 36215360

[30] Khanna, R., Guler, I. & Nerkar, A. Fail often, fail big, and fail fast? Learning from small failures and R &D performance in the pharmaceutical industry. *Acad. Manag. J.* **59**(2), 436–459 (2016).

[31] Jordan, D. An overview of the common technical document (CTD) regulatory dossier. *Med Writ.* **23**(2), 101–105 (2014).

[32] Bois, F. et al. [68Ga] Ga-PSMA-11 in prostate cancer: A comprehensive review. *Am. J. Nucl. Med. Mol. Imaging* **10**(6), 349 (2020). 33329937 PMC7724278

[33] Piron, S., Verhoeven, J., Vanhove, C. & De Vos, F. Recent advancements in 18F-labeled PSMA targeting PET radiopharmaceuticals. *Nucl. Med. Biol.* **106**, 29–51 (2022). 34998217 10.1016/j.nucmedbio.2021.12.005

[34] Collet, C. et al. Fully automated production of sodium [18F] fluoride on AllInOne and miniAllInOne synthesizers. *Appl. Radiat. Isotopes* **102**, 87–92 (2015). 10.1016/j.apradiso.2015.04.016 26002274

[35] Bastogne, T., Caputo, F., Prina-Mello, A., Borgos, S., & Barberi-Heyob, M. A state of the art in analytical quality-by-design and perspectives in characterization of nano-enabled medicinal products. *J. Pharmaceut. Biomed. Anal.* **219**(114911) (2022) 10.1016/j.jpba.2022.114911 35779356

[36] ICH Harmonised Tripartite Guideline: Pharmaceutical Development Q8(R2). Current Step 4 Version, International Conference on Harmonisation of Technical Requirements for Registration of Pharmaceuticals for Human Use (2009).

[37] Wang, H. & Yeung, D.-Y. Towards Bayesian deep learning: A framework and some existing methods. *IEEE Trans. Knowl. Data Eng.* **28**(12), 3395–3408 (2016).

[38] Wang, H. & Yeung, D.-Y. A survey on Bayesian deep learning. *ACM Comput. Surv. (CSUR)* **53**(5), 1–37 (2020).

[39] Abdullah, A. A., Hassan, M. M. & Mustafa, Y. T. A review on Bayesian deep learning in healthcare: Applications and challenges. *IEEE Access* **10**, 36538–36562 (2022).

