# Supplementary material for: A hybrid innovation method based on quality by design and agile scrum paradigms for the development of medicinal products
Source: Sci Rep. 2025 Sep 29;15:33666. doi: 10.1038/s41598-025-18181-w (PMC12479877; doi:10.1038/s41598-025-18181-w)
Supplement: Supplementary file 1 — Supplementary Information. [file 41598_2025_18181_MOESM1_ESM.pdf]

Supplementary File: A Hybrid Innovation Method based on Quality by Design and Agile Scrum paradigms for the Development of Medicinal Products

Thierry Bastogne[1*], Laurène Wagner[2,3], Samir Acherar[2], Gilles Karcher[3], Charlotte Collet[3,4]

[1*]CRAN UMR 7039, Université de Lorraine, CNRS, Nancy, 54000, France.
[2]LCPM UMR 7375, Université de Lorraine, CNRS, Nancy, 54000, France.
[3]Nancyclotep, Vandoeuvre-lès-Nancy, 54511, France.
[4]IADI, Université de Lorraine, INSERM U1254, Vandoeuvre-lès-Nancy, 54511, France.

*Corresponding author(s). E-mail(s): thierry.bastogne@univ-lorraine.fr;

Abstract

This study proposes an agile paradigm of the pharmaceutical Quality by Design (QbD) approach initiated and recommended by regulatory agencies to better understand and control their innovative products and processes throughout the development phase. The primary objective of this hybrid innovation method is to improve the structural organization of the QbD approach to simplify its use and expand its application to the early stages of preclinical development. This agile QbD paradigm relies on the incrementation and/or iteration of short studies called sprints indexed according to the Technological Readiness Level (TRL) scale. Each QbD sprint addresses a priority question of development and relies on a hypothetico-deductive scientific method to address it. They are composed of five steps: developing and updating the Target Product Profile, identifying critical input and output variables, designing experiments, conducting experiments, and analyzing the collected data to generalize conclusions through statistical inference. At the end of a sprint, four outcomes are possible: incrementing knowledge on the developed drug, i.e. moving to the next development sprint, iterating the current or previous sprint to reduce decision-making risk,

pivoting to propose a new product profile, or stopping the development project. This decision-making process is based on the results of a statistical analysis estimating the probability of meeting the efficacy/safety/quality specifications of the medicinal product to be developed. To illustrate and to assess the practical relevance of this incremental and iterative approach, we applied it to the development of a new radiopharmaceutical for Positron Emission Tomography (PET) imaging. The Agile QbD approach was applied over six consecutive sprints to progress from an initial product concept (TRL 2) to a prototype manufactured using a production automation system (TRL 4). The method and results presented in this study provide a new perspective on applying QbD as an efficient tool for managing knowledge during innovation projects.

Keywords: Quality by Design, Agile method, Data-driven innovation, Radiopharmaceutical

1 Main steps of the radiopharmaceutical manufacturing process

Figure SF1 presents the three main stages of the radiopharmaceutical manufacturing process.

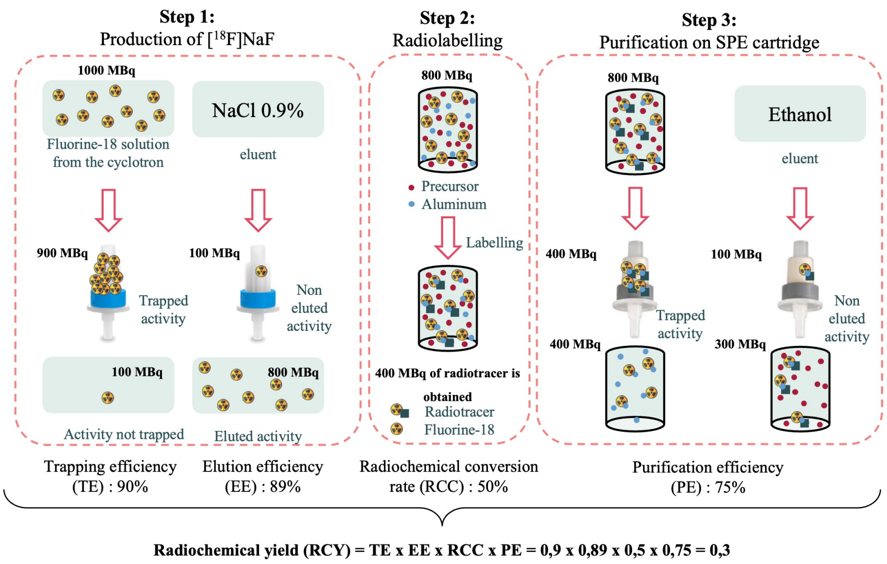

Fig. SF1 Main steps of the radiopharmaceutical manufacturing.

2 Details of results in sprint $S1.1$

Sprint $S1$ was focused on the objective to identify the most critical input variables by implementing a data-driven modeling approach. The investigation model used to solve this factor screening issue is defined as follows:

$$Y = b_0 + b_1 u_1 + b_2 u_2 + b_3 u_3 + b_4 u_4 + b_5 u_5 + b_6 u_6 + b_7 u_7 + E, \tag{1}$$

where Y denotes the radiochemical conversion ratel, u_i is the i-th input variable to be tested, and $E \sim \mathcal{N}(0, \sigma^2)$. The coefficients b_i of the model allow us to assess the impact strength of each associated input variable. The larger their absolute value, the more critical the corresponding input factor is for Y. These coefficients directly help us answer the screening question by identifying the variables whose coefficients are considered the most active. The coefficients b_i are initially unknown but have been estimated from a dataset obtained after conducting during pilot tests. Table 2 shows the content of the initial dataset. It is composed of 30 experiments during which the temperature $u8$ was maintained constant at $50°C$ during all the assays except the last one. In fact, this factor is already known as critical and can thus be excluded from the screening. This is the reason it does not appear in the investigation model (1).

Figure SF2 presents the correlation matrix for the seven inputs to be tested. A strong correlation between u_3 and u_4 is detected. As a consequence, only one (u_3) of the two was kept for the estimation step.

Table SF1 Dataset of the first QbD ($S1$) Sprint applied to the screening of critical input factors

	u_1	u_2	u_3	u_4	u_5	u_6	u_7	u_8	Y
1	20.00	0.80	50.00	11.60	23.10	4320.00	15.00	50	26.81
2	20.00	0.80	50.00	11.60	23.10	4320.00	15.00	50	0.66
3	25.00	2.50	20.00	8.70	43.60	2292.00	15.00	50	8.60
4	25.00	2.50	20.00	9.80	36.70	2045.00	15.00	50	4.62
5	25.00	2.50	20.00	8.70	43.60	2295.00	15.00	50	27.00
6	25.00	2.50	20.00	8.70	43.60	2295.00	15.00	50	20.90
7	25.00	2.50	20.00	8.70	43.60	2295.00	15.00	50	11.48
8	25.00	2.50	20.00	5.60	42.30	3545.00	15.00	50	18.10
9	10.00	1.00	20.00	6.60	49.50	3030.00	15.00	50	9.82
10	10.00	1.00	20.00	6.60	33.00	3030.00	10.00	50	17.14
11	8.00	0.80	20.00	6.60	33.00	3028.00	10.00	50	9.84
12	12.00	1.20	20.00	6.60	33.00	3032.00	10.00	50	3.00
13	8.00	0.80	20.00	6.60	33.00	3028.00	10.00	50	4.14
14	10.00	1.00	20.00	5.80	35.00	3430.00	10.00	50	5.44
15	10.00	1.00	20.00	6.60	49.50	3030.00	10.00	50	25.32
16	10.00	0.57	35.00	11.50	49.30	3045.00	10.00	50	9.62
17	37.50	1.00	35.00	11.40	48.80	3072.50	10.00	50	3.19
18	8.00	0.80	20.00	6.60	49.50	3028.00	10.00	50	21.60
19	25.00	1.00	50.00	16.30	48.80	3075.00	10.00	50	30.70
20	12.00	1.20	20.00	6.60	49.50	3032.00	10.00	50	13.06
21	25.00	1.00	50.00	16.30	32.50	3075.00	15.00	50	32.96
22	34.00	0.80	85.00	27.30	54.50	3119.00	15.00	50	86.04
23	20.00	0.80	50.00	24.20	55.60	2070.00	15.00	50	49.32
24	25.00	1.00	50.00	24.10	22.90	2075.00	15.00	50	7.59
25	20.00	0.80	50.00	24.20	55.60	2070.00	15.00	50	28.39
26	15.00	0.60	50.00	24.20	55.70	2065.00	15.00	50	76.94
27	10.00	0.40	50.00	24.30	55.80	2060.00	15.00	50	43.88
28	10.50	0.60	35.00	17.10	56.20	2045.50	15.00	50	44.69
29	15.00	0.60	50.00	24.20	55.70	2065.00	15.00	50	36.31
30	18.00	0.60	60.00	28.90	55.30	2078.00	20.00	20	53.00

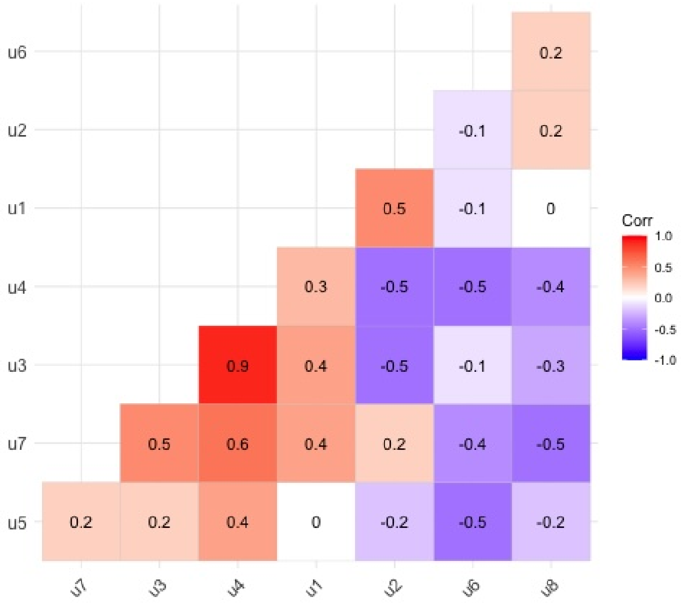

Fig. SF2 Correlation matrix for the seven input variables involved in the sprint $S1$

3 Details of results in sprint $S2.1$

Sprint $S2$ was focused on the objective to identify the optimal operating region within the space of the four inputs: (u_2, u_3, u_5, u_8), *i.e.* a subspace in which each operating point likely meets the specification on Y. The DoE and responses measured during the experiments are presented in the Table 3. A stepwise regression was applied to keep only the relevant terms in the model. Since Y was measured by two different protocols: UPLC and CCM, two models were determined: Y_1 for the UPLC data and Y_2 for the CCM data. The final models is defined as follows:

$$Z_1 = b_0 + b_2\,u_2 + b_3\,u_3 + b_5\,u_5 + b_8\,u_8 + b_{55}\,u_5^2 + b_{25}\,u_2u_5 + b_{38}\,u_3u_8 + b_{58}\,u_5u_8 + E_1 \quad (2)$$

$$Z_2 = b_0 + b_2\,u_2 + b_3\,u_3 + b_5\,u_5 + b_8\,u_8 + b_{33}\,u_3^2 + b_{55}\,u_5^2 + b_{28}\,u_2u_8 + b_{58}\,u_5u_8 + E_2, \quad (3)$$

with $E_1 \sim \mathcal{N}(0, \sigma_1^2)$ and $E_1 \sim \mathcal{N}(0, \sigma_2^2)$. Their coefficients of determination are respectively: $R_1^2 \approx 0.814$ and $R_2^2 \approx 0.793$. The estimated values of the coefficients for the two models are given in Tables SF3 and SF4.

Table SF2 Dataset of the second QbD Sprint ($S2$) applied to the identification of an optimal operating region. Y_1 and Y_2 denote the radiochemical conversion rate measured by UPLC and CCM protocols respectively.

	u_3	u_2	u_8	u_5	Y_1	Y_2
1	20.00	0.50	65.00	20.00	4.22	7.32
2	80.00	0.50	65.00	20.00	29.33	29.08
3	20.00	1.50	35.00	20.00	1.79	5.38
4	80.00	1.50	35.00	20.00	2.98	7.24
5	20.00	0.50	35.00	80.00	63.04	84.60
6	80.00	0.50	35.00	80.00	63.84	85.69
7	20.00	1.50	65.00	80.00	3.95	4.77
8	80.00	1.50	65.00	80.00	19.83	7.48
9	20.00	1.00	50.00	50.00	39.15	47.59
10	80.00	1.00	50.00	50.00	62.36	83.27
11	50.00	0.50	50.00	50.00	71.37	93.87
12	50.00	1.50	50.00	50.00	19.61	29.80
13	50.00	1.00	50.00	20.00	22.13	19.67
14	50.00	1.00	50.00	80.00	56.77	94.20
15	50.00	1.00	35.00	50.00	78.91	93.41
16	50.00	1.00	65.00	50.00	60.33	73.42
17	50.00	1.00	50.00	50.00	54.92	89.29
18	20.00	0.50	65.00	20.00	5.69	4.41
19	80.00	0.50	65.00	20.00	38.30	33.27
20	20.00	1.50	35.00	20.00	2.61	17.39
21	80.00	1.50	35.00	20.00	9.10	28.79
22	20.00	0.50	35.00	80.00	70.32	93.82
23	80.00	0.50	35.00	80.00	76.36	93.26
24	20.00	1.50	65.00	80.00	47.66	69.60
25	80.00	1.50	65.00	80.00	68.58	85.44
26	20.00	1.00	50.00	50.00	32.50	37.96
27	80.00	1.00	50.00	50.00	42.69	72.28
28	50.00	0.50	50.00	50.00	73.35	93.33
29	50.00	1.50	50.00	50.00	17.61	25.93
30	50.00	1.00	50.00	20.00	4.03	5.97
31	50.00	1.00	50.00	80.00	72.20	91.89
32	50.00	1.00	35.00	50.00	43.91	67.02
33	50.00	1.00	65.00	50.00	21.55	26.52
34	50.00	1.00	50.00	50.00	53.75	78.07
35	80.00	0.50	35.00	80.00	58.25	87.50
36	20.00	1.50	65.00	80.00	38.28	51.12
37	80.00	1.50	65.00	80.00	61.48	80.65
38	80.00	1.00	50.00	50.00	41.22	70.75
39	50.00	1.00	50.00	20.00	5.03	11.60
40	50.00	1.00	35.00	50.00	28.09	57.02
41	50.00	1.00	65.00	50.00	15.17	22.71
42	50.00	1.00	50.00	50.00	44.72	66.59
43	65.00	0.75	50.00	80.00	84.01	91.39
44	65.00	0.75	40.00	65.00	89.59	96.86
45	80.00	0.50	60.00	50.00	91.08	98.60
46	65.00	0.75	50.00	80.00	78.70	99.00
47	50.00	1.00	40.00	50.00	77.89	90.21
48	80.00	0.75	50.00	65.00	82.17	90.81
49	65.00	0.50	50.00	65.00	89.09	95.82
50	65.00	0.75	40.00	65.00	92.26	99.00
51	50.00	0.75	50.00	65.00	84.27	96.92

Table SF3 Estimated values of the model coefficients for the output Z_1 (UPLC), Sprint $S2$.

	Estimate	Std. Error
b0	7.2104	3.8769
b2	-5.0724	1.4245
b3	-0.0163	0.0213
b5	0.0323	0.0593
b8	-0.1689	0.0525
b55	-0.0012	0.0003
b25	0.0361	0.0225
b38	0.0007	0.0004
b58	0.0020	0.0008

Table SF4 Estimated values of the model coefficients for the output Z_2 (CCM), Sprint $S2$.

	Estimate	Std. Error
b0	-1.1956	5.9701
b2	2.7351	3.4611
b3	0.0785	0.0404
b5	0.0345	0.0737
b8	-0.0552	0.0953
b33	-0.0006	0.0004
b55	-0.0010	0.0004
b28	-0.1322	0.0619
b58	0.0029	0.0011

4 Details of results in sprint $S3.1$

Table SF5 presents the Fractional Factorial Design and associated dataset related to the third QbD Sprint ($S3$).

Table SF5 2^{4-1} Fractional Factorial Design and associated dataset related to the third QbD Sprint ($S3$), used to qualify an hypercubic operating region. Y_1 and Y_2 denote the radiochemical conversion rate measured by UPLC and CCM protocols respectively.

No.	u3	u2	u8	u5	Y1	Y2
1	67	0.50	62	68	88.08	95.36
2	80	0.50	62	80	84.63	93.81
3	67	0.90	62	80	82.72	91.11
4	80	0.90	62	68	87.72	98.85
5	67	0.50	65	80	84.48	94.42
6	80	0.50	65	68	85.10	91.42
7	67	0.90	65	68	84.64	96.45
8	80	0.90	65	80	86.02	94.02

5 Details of results in sprint $S4.1$

Sprint $S4.1$ was focused on the objective to identify the optimal operating region within the space of the four inputs: (u_2, u_3, u_5, u_8), *i.e.* a subspace, called design space, in which each operating point likely meets the specification on Y with an acceptable probability. To solve this problem the same data-driven modeling approach implemented in $S2$ was used. The resulting models are defined as follows:

$$Z_1 = b_0 + b_2\, u_2 + b_5\, u_5 + b_{22}\, u_2^2 + b_{88}\, u_8^2 + b_{25}\, u_2 u_5 + b_{28}\, u_2 u_8 + b_{35}\, u_3 u_5 + b_{58}\, u_5 u_8 + E_1 \quad (4)$$

$$Z_2 = b_0 + b_3\, u_3 + b_5\, u_5 + b_{22}\, u_2^2 + b_{88}\, u_8^2 + b_{23}\, u_2 u_3 + b_{25}\, u_2 u_5 + b_{38}\, u_3 u_8 + b_{58}\, u_5 u_8 + E_2, \quad (5)$$

with $E_1 \sim \mathcal{N}(0, \sigma_1^2)$ and $E_1 \sim \mathcal{N}(0, \sigma_2^2)$. Their coefficients of determination are respectively: $R_1^2 \approx 0.94$ and $R_2^2 \approx 0.971$. The estimated values of the coefficients for the two models are given in Tables SF7 and SF8.

Table SF6 Hartley Composite Design and associated dataset related to the fourth QbD Sprint ($S4$). Y_1 and Y_2 denote the radiochemical conversion rate measured by UPLC and CCM protocols respectively.

u3	u2	u5	u8	Y1	Y2	Z1	Z2
20	0.50	20.00	65	0.77	0.73	-4.86	-4.91
20	1.00	50.00	50	36.72	41.79	-0.54	-0.33
20	1.50	20.00	35	4.38	4.62	-3.08	-3.03
20	0.50	80.00	35	21.08	38.27	-1.32	-0.48
20	1.50	80.00	65	50.56	79.64	0.02	1.36
50	1.50	50.00	50	14.40	31.92	-1.78	-0.76
50	1.00	50.00	65	17.09	26.08	-1.58	-1.04
50	1.00	20.00	50	5.74	7.00	-2.80	-2.59
50	1.00	50.00	50	18.78	49.66	-1.46	-0.01
50	1.00	50.00	50	44.89	56.40	-0.21	0.26
50	1.00	50.00	50	23.04	28.57	-1.21	-0.92
50	1.00	50.00	50	44.04	53.63	-0.24	0.15
50	1.00	50.00	50	54.54	59.62	0.18	0.39
50	1.00	50.00	35	51.51	72.38	0.06	0.96
50	1.00	80.00	50	97.00	93.91	3.48	2.74
50	0.50	50.00	50	30.60	38.00	-0.82	-0.49
80	0.50	80.00	50	68.09	73.11	0.76	1.00
80	0.50	20.00	65	1.19	1.07	-4.42	-4.53
80	1.50	80.00	65	74.03	97.28	1.05	3.58
80	1.00	50.00	50	53.98	75.94	0.16	1.15
80	0.50	80.00	35	50.50	40.50	0.02	-0.38
80	1.50	20.00	35	2.78	6.06	-3.55	-2.74
75	1.40	75.00	55	95.80	93.80	3.13	2.72
75	1.40	75.00	55	56.70	66.00	0.27	0.66
75	1.40	75.00	55	50.30	47.60	0.01	-0.10
80	1.40	80.00	55	43.90	74.40	-0.25	1.07
80	1.40	80.00	55	64.90	45.50	0.61	-0.18
80	1.10	75.00	55	65.50	89.10	0.64	2.10
80	1.10	75.00	55	68.90	75.80	0.80	1.14
75	1.40	80.00	40	30.50	44.20	-0.82	-0.23
75	1.40	80.00	40	56.60	57.30	0.27	0.29
75	1.40	80.00	40	42.50	35.10	-0.30	-0.61
75	1.10	80.00	55	69.10	67.70	0.80	0.74
75	1.10	80.00	55	70.70	55.20	0.88	0.21
80	1.10	80.00	40	30.50	44.20	-0.82	-0.23
80	1.10	80.00	40	35.90	24.70	-0.58	-1.11
75	1.10	75.00	40	9.10	8.20	-2.30	-2.42
80	1.40	75.00	40	22.90	33.10	-1.21	-0.70
80	1.40	75.00	40	34.40	33.90	-0.65	-0.67
75	1.10	75.00	45	79.50	90.30	1.36	2.23
75	1.10	75.00	48	81.10	92.30	1.46	2.48
75	1.40	80.00	45	51.20	37.00	0.05	-0.53
75	1.40	80.00	50	39.90	39.90	-0.41	-0.41
80	1.40	75.00	45	64.40	61.50	0.59	0.47
80	1.40	75.00	50	54.40	56.80	0.18	0.27
75	0.80	80.00	55	63.50	64.20	0.55	0.58
75	0.80	80.00	55	56.60	63.80	0.27	0.57
75	0.80	80.00	50	46.10	44.80	-0.16	-0.21

Table SF7 Estimated values of the model coefficients for the output Z_1 (UPLC), Sprint $S4.1$.

	Estimate	Std. Error
b0	-4.1789	3.8091
b2	12.4192	4.3775
b5	-0.1117	0.0566
b22	-5.6278	1.1062
b88	-0.0008	0.0007
b25	0.0692	0.0279
b28	-0.1117	0.0558
b35	0.0002	0.0001
b58	0.0025	0.0009

Table SF8 Estimated values of the model coefficients for the output Z_2 (CCM), Sprint $S4.1$.

	Estimate	Std. Error
b0	5.0509	1.2910
b3	-0.0343	0.0206
b5	-0.1754	0.0333
b22	-3.0913	0.4528
b88	-0.0027	0.0003
b23	0.0185	0.0101
b25	0.0933	0.0161
b38	0.0006	0.0003
b58	0.0033	0.0006

6 Details of results in sprints $S5.1$ and $S5.2$

A first sprint $S5.1$ was applied to qualify the design space identified in the sprint $S4.1$. The design of experiments implemented in $S5.1$ is presented in Table SF9. To complete the results obtained during this sprint, we had to run a second iteration $S5.2$ of the sprint. Its associated experimental design is given in Table SF10.

Table SF9 2^{4-1} Fractional Factorial design and associated dataset related to the fifth QbD Sprint ($S5.1$). Y_1 and Y_2 denote the radiochemical conversion rate measured by UPLC and CCM protocols respectively.

No.	u3	u2	u5	u8	Y1	Y2
1	75	1,4	75	55	95,8	93,80
2	80	1,4	80	55	76,1	74,80
3	80	1,1	75	55	92,1	97,00
4	75	1,4	80	40	48,8	44,20
5	75	1,1	80	55	95,5	78,50
6	80	1,1	80	40	83,5	72,60
7	75	1,1	75	40	10	9,00
8	80	1,4	75	40	46,8	33,00

Table SF10 Complementary design of experiments related to the QbD Sprint ($S5.2$). Y_1 and Y_2 denote the radiochemical conversion rate measured by UPLC and CCM protocols respectively.

No.	u3	u2	u8	u5	Y1	Y2
1	75	1.10	40	70	33	35
2	75	1.10	40	80	89	86
3	75	1.00	40	75	89	89
4	75	1.20	40	75	69	78
5	75	1.10	45	75	71	75
6	70	1.10	40	75	71	74
7	80	1.10	40	75	76	83
8	75	1.10	35	75	63	61
